# Port-based teleportation in arbitrary dimension

**DOI:** 10.1038/s41598-017-10051-4

**Published:** 2017-09-07

**Authors:** Michał Studziński, Sergii Strelchuk, Marek Mozrzymas, Michał Horodecki

**Affiliations:** 1 DAMTP, Centre for Mathematical Sciences, University of Cambridge, Cambridge, CB30WA UK; 2 Institute for Theoretical Physics, University of Wrocław, 50-204 Wrocław, Poland; 3 Institute of Theoretical Physics and Astrophysics, National Quantum Information Centre, Faculty of Mathematics, Physics and Informatics, Univeristy of Gdańsk, Wita Stwosza 57, 80-308 Gdańsk, Poland

## Abstract

Port-based teleportation (PBT), introduced in 2008, is a type of quantum teleportation protocol which transmits the state to the receiver without requiring any corrections on the receiver’s side. Evaluating the performance of PBT was computationally intractable and previous attempts succeeded only with small systems. We study PBT protocols and fully characterize their performance for arbitrary dimensions and number of ports. We develop new mathematical tools to study the symmetries of the measurement operators that arise in these protocols and belong to the algebra of partially transposed permutation operators. First, we develop the representation theory of the mentioned algebra which provides an elegant way of understanding the properties of subsystems of a large system with general symmetries. In particular, we introduce the theory of the partially reduced irreducible representations which we use to obtain a simpler representation of the algebra of partially transposed permutation operators and thus explicitly determine the properties of any port-based teleportation scheme for fixed dimension in polynomial time.

## Introduction

Quantum teleportation is one of the most important primitives in the Quantum Information Processing^1^. This technique allows to transfer the state of an unknown quantum system from the sender to the receiver without having to exchange the physical system. It has led to a large number of theoretical advances in quantum information theory and quantum computing^2–9^.

The first teleportation protocol involved two parties, Alice and Bob, each sharing a half of the maximally entangled state^1^. We will further refer to it as a ‘resource state’. Alice wants to send an (unknown) state of a subsystem in her possession to Bob. She performs a projective measurement on her subsystem and the half of the maximally entangled state and communicates its classical outcome to Bob. He then reliably recovers the state which Alice communicated by applying a unitary correction operation conditioned on Alice’s message.

In 2008, a breakthrough result from Ishizaka and Hiroshima introduced a novel port-based teleportation protocol (PBT) which does not require the last step in the sequence^10^. In this setup, parties share a large resource state consisting of *N* copies of the maximally entangled states $|{{\rm{\Psi }}}^{-}{\rangle }^{\otimes N}$, where each singlet is a two-qubit state, termed *port*. Alice performs a joint measurement ${\mathscr{X}}$ on the unknown state *θ* which she wishes to teleport and her half of the resource state, communicating the outcome to Bob. The outcome of the measurement points to the subsystem where the state has been teleported to. To obtain the teleported state Bob discards all ports except for the one indicated by Alice’s outcome. There are two versions of the PBT protocol, depending on the exact set of measurements used by Alice. The first type, so-called *deterministic* teleportation, is described by the set of *N* POVM elements ${\mathscr{X}}={\{{{\rm{\Pi }}}_{a}\}}_{a=1}^{N}$. Upon measuring *a*-th element the teleported state ends up in the *a*-th port on Bob’s side. He then traces out all but *a*-th subsystem which contains the teleported state. The second type, *probabilistic* PBT, consists of a measurement with *N* + 1 POVM elements ${\{{{\rm{\Pi }}}_{a}\}}_{a=0}^{N}$, where ${{\rm{\Pi }}}_{0}$ indicates a failure of the teleportation. In this protocol, when Alice obtains the input $a\in \mathrm{\{1,}\ldots ,N\}$, the parties proceed as above. When she obtains 0, then they abort the protocol.

In the probabilistic PBT the state of a qubit always gets teleported to Bob, but it decoheres during the process. The lower bound on the fidelity of the teleported state tends to 1 as the number of ports $N\to \infty$. In the deterministic case protocol, the state always gets teleported to Bob with perfect fidelity, but with some probability (which vanishes in the limit $N\to \infty$) Alice aborts.

PBT schemes found novel applications in the areas where the existing teleportation schemes fell short of. They provided new architecture for the universal programmable quantum processor performing computation by teleportation with the property of it being composable^11^. In position-based cryptography, PBT schemes were used to engineer efficient protocols for instantaneous implementation of measurement and computation. It resulted in new attacks on the cryptographic primitives, reducing the amount of consumable entanglement from doubly exponential to exponential^12^.

Recently, the composable nature of the qubit PBT schemes made it possible to connect the field of communication complexity and a Bell inequality violation^13^. It allowed to show that any quantum advantage obtained by a protocol for an arbitrary communication complexity problem resulted in the violation of a Bell inequality, certifying the quantum nature of the advantage.

The full characterization of the qubit PBT schemes was used to obtain the performance of the square-root measurements for mixed states obtaining explicit probabilities of success when the set of states to be discriminated has certain symmetries and POVMs are of nearly maximal rank^12^.

Evaluating the performance of the PBT is tantamount to determining the spectral properties of the measurement operators ${\mathscr{X}}$. To determine them, authors in ref. 10 viewed *N* + 1 qubits (with one extra qubit representing the teleported state) as spins, recursively building a basis for constituents of ${\mathscr{X}}$ making the use of the Clebsch-Gordan (CG) coefficients and with the painstaking amount of effort determined their eigenvalues. This approach has been successful for studying systems of *N* + 1 qubits and relied on the existence of the closed form for the CG coefficients and therefore was limited to $SU{\mathrm{(2)}}^{\otimes N}$. In the case of $SU{(d)}^{\otimes N}$, with *d* > 2 there exists no closed form of the CG coefficients and thus it is impossible to obtain the spectrum of ${\mathscr{X}}$ without incurring an exponential overhead in *d* and *N*. It is however possible to obtain a closed-form lower bound on the performance of deterministic PBT, but it only works in the regime $N\gg d$. Moreover, no bound is known for the probabilistic PBT.

By using graphical variant of Temperley-Lieb algebra, authors obtained an explicit closed-form expressions for the fidelity and success probability of PBT for an arbitrary *d* and $N\in \mathrm{\{2,}\,\mathrm{3,}\,\mathrm{4\}}$ ^14^. Here too the mathematical formulae contain the number of different terms which grows exponentially in *N*.

In our work we develop new mathematical tools to study the symmetries of ${\mathscr{X}}$ which enable us to efficiently evaluate the performance of *any* PBT scheme for arbitrary *N* and *d*. Our first contribution is the theory of the partially reduced irreducible representations (PRIR). They provide an elegant way of understanding the properties of subsystems of a large system which has general symmetries. We further use these techniques to provide a simple way to approach to the representation of the algebra of the partially transposed permutation operators. Remarkably, the operators describing measurements in any PBT scheme possess the exact symmetries of an element of this algebra. By exploring these symmetries in a principled way we are able to fully analyze all teleportation schemes.

We thus characterize the performance of the main PBT schemes and find exact expressions for the fidelity of the teleportation and the probability of success in the deterministic and probabilistic schemes respectively. Moreover, we describe the spectral properties of the POVMs and exhibit polynomial algorithms to efficiently calculate the properties of quantum systems with similar symmetries using our framework.

## Setting and Main Results

In this section we introduce the setting and outline the results obtained by new mathematical techniques developed in our work.

Consider a probabilistic protocol defined by a *d*-dimensional maximally entangled resource state ${\otimes }_{i=1}^{N}|{{\rm{\Phi }}}_{d}^{+}{\rangle }_{{A}_{i}{B}_{i}}$, set of POVMs ${\mathscr{X}}={\{{{\rm{\Pi }}}_{a}\}}_{a=0}^{N}$, where each ${{\rm{\Pi }}}_{a}={\rho }^{-\frac{1}{2}}{\rho }_{a}{\rho }^{-\frac{1}{2}}$ for $a\ge 1$ and ${{\rm{\Pi }}}_{0}={\bf{1}}-{\sum }_{a=1}^{N}{{\rm{\Pi }}}_{a}$ with

1 $$\rho =\sum _{a=1}^{N}{\rho }_{a},$$

and $${\rho }_{a}=\frac{1}{{d}^{N}}{P}_{C{A}_{a}}^{+}\otimes {{\bf{1}}}_{\overline{{A}_{a}}}$$, for $$a=1,\ldots ,N$$.

**Figure 1:**
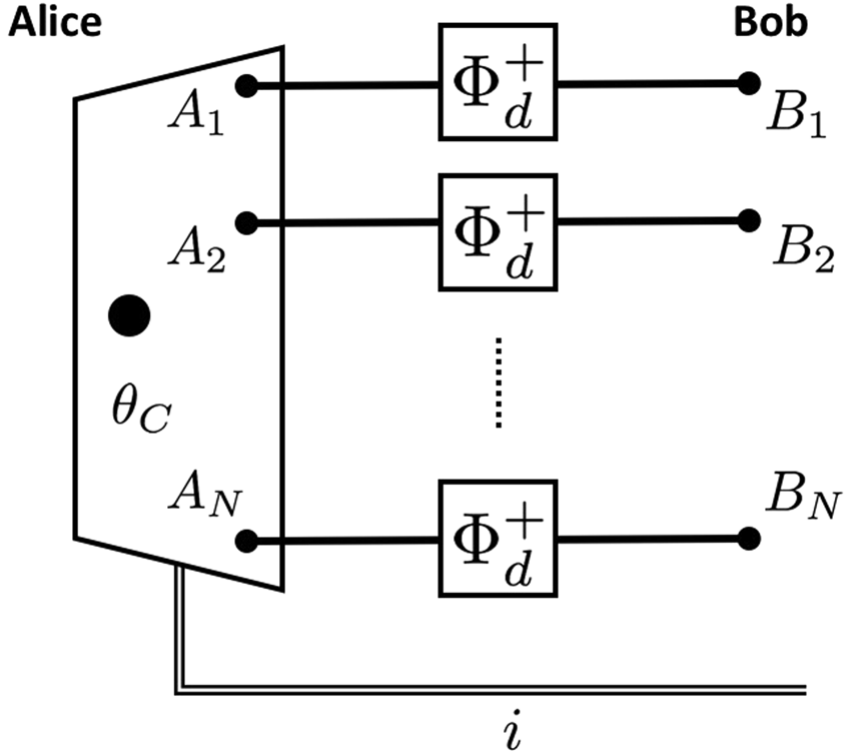
Schematic description of PBT in the arbitrary dimension.

The operator ${P}_{C{A}_{a}}^{+}$ denotes an unnormalised projection onto the state $|{{\rm{\Phi }}}^{+}{\rangle }_{C{A}_{a}}={\sum }_{i=1}^{d}|{\rm{i}}{\rm{i}}{\rangle }_{C{A}_{a}}$ between systems *C* and *A*_*a*_, and ${{\bf{1}}}_{\overline{{A}_{a}}}$ is identity operator on all subsystems *A* except *A*_*a*_. We will henceforth refer to *ρ* as the PBT operator. Alice wishes to teleport a qudit *θ*_*C*_ to Bob. After she measures ${\mathscr{X}}$ and communicates the classical outcome *i* to Bob, he performs one of the following actions: (a) if $i\in \mathrm{\{1,}\ldots ,N\}$ he traces out all but the *i*-th port which contains the teleported state with perfect fidelity; (b) if *i* = 0 he aborts. The schematic representation is shown in Fig. 1.

### Eigenvalues of the PBT operator

We develop new mathematical tools and find that the PBT operator has the following spectrum:

**Proposition 2**. [Short version] *The eigenvalues of the PBT operator ρ in* *Eqn*. (1) *are given by* ${\lambda }_{\mu }(\alpha )=\frac{N}{{d}^{N}}\frac{{m}_{\mu }{d}_{\alpha }}{{m}_{\alpha }{d}_{\mu }}$. *Quantities* ${m}_{\alpha },{m}_{\mu }$ *denote multiplicities in the natural representation*, ${d}_{\alpha },{d}_{\mu }$ *denote the respective dimensions of the irreps*. *By μ we denote Young diagrams obtained from Young diagrams α of* n − 2 *boxes by adding a single box in a proper way*. *We take Young diagrams* α, μ *whose height is not greater then dimension d of the local Hilbert space*.

Further in this manuscript by symbol $\nu \, \vdash \,m$ we denote Young diagram of *m* boxes, by $\mu \in \alpha$ we denote all Young diagrams *μ* which can be obtained from the Young diagram *α* by adding a single box in a proper way, $\alpha \in \mu$ denotes all Young diagrams *α* which can be obtained from the Young diagram *μ* by removing a single box. By ‘proper way’ we understand a situation when the height of final Young diagram is less or equal than dimension *d* of the local Hilbert space. For the Young diagram *μ* its height (number of rows) is denoted as *h*(*μ*). Since there is one-to-one correspondence between Young diagrams made up of *n* boxes and inequivalent irreps of the symmetric group *S*(*n*) we use symbols *α*, *μ* etc. interchangeably for Young diagrams and irreps whenever it is clear from the context.

### Probabilistic PBT

In this scheme the teleported state reaches the recipient with high probability and one distinguishes two different protocols each with perfect teleportation fidelity. In the first type we consider a resource state which consists of a number of maximally entangled states, and in the second type we obtain the resource state as well set of POVMs as a result of the optimization procedure. In the former case, the probability of success is given by

**Theorem 3**. *The maximal average success probability in the probabilistic PBT with a resource state consisting of maximally entangled pairs is given by* $p=\frac{1}{{d}^{N}}{\sum }_{\alpha }{m}_{\alpha }^{2}{{\rm{\min }}}_{\mu \in \alpha }\frac{{d}_{\mu }}{{m}_{\mu }}$, *where μ denotes Young diagram obtained from Young diagrams* $\alpha  \vdash n-2$ *by adding a single box in a proper way and* ${\gamma }_{\mu }(\alpha )$ *is given in Proposition* 2.

In the latter case, the probability of success is given by

**Theorem 4**. *The optimal state in the probabilistic PBT is given by* ${X}_{A}={\sum }_{\mu }{c}_{\mu }{P}_{\mu }\quad {\rm{with}}\quad {c}_{\mu }=\frac{{d}^{N}g(N){m}_{\mu }}{{d}_{\mu }}$ *where* $g(N)=1/{\sum }_{\nu }{m}_{\nu }^{2}$, *and* $\nu$ *labels irreps of* $S(n-\mathrm{1)}$. *Operators* ${P}_{\mu }$ *are Young projectors onto irreps of* $S(n-\mathrm{1)}$. *The corresponding optimal probability is of the form* $p=1-\frac{{d}^{2}-1}{N+{d}^{2}-1}$, *where N is the number of ports*, *and d is the dimension of the local Hilbert space*.

**Figure 2:**
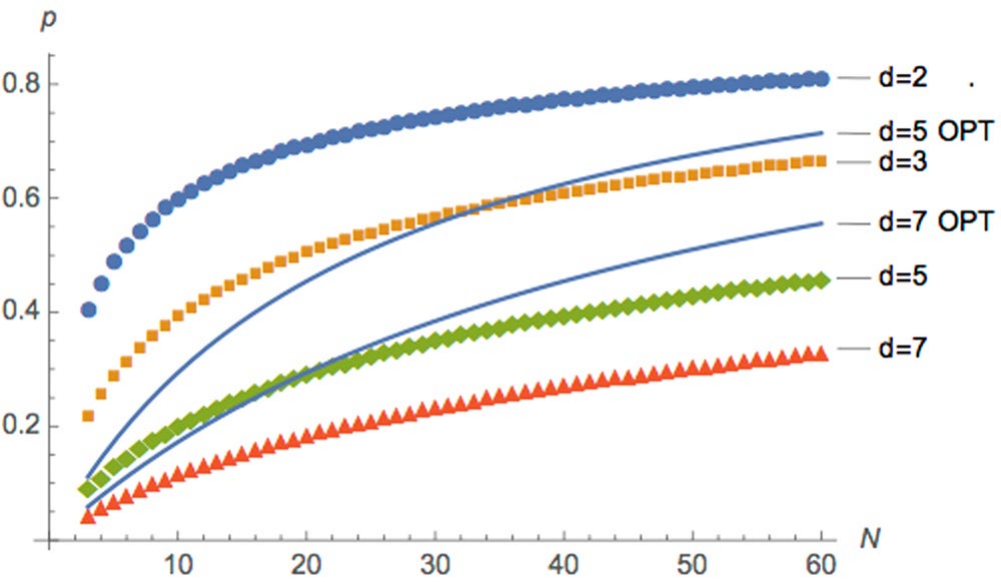
Exact performance of the probabilistic PBT protocol. Dotted lines correspond to the average success probability when we only optimize the measurements using maximally entangled resource state. Solid lines correspond to the average success probability when we optimize both the measurement and the resource state.

Figure 2 depicts the success probability computed by our algorithm for both cases.

### Deterministic PBT

The deterministic version of the PBT guarantees that the teleported state always reaches the recipient, but at a cost of being distorted. It is described by *N* POVM elements ${\{{{\rm{\Pi }}}_{a}\}}_{a=1}^{N}$ with each ${{\rm{\Pi }}}_{a}={\rho }^{-\mathrm{1/2}}{\rho }_{a}{\rho }^{-\mathrm{1/2}}+{\rm{\Delta }}$, where the term ${\rm{\Delta }}=\mathrm{(1}/N)({\bf{1}}-{\sum }_{a=1}^{N}{{\rm{\Pi }}}_{a})$ with $\mathrm{Tr}{\rho }_{a}{\rm{\Delta }}=0$ is required to ensure that ${\sum }_{a=1}^{N}{{\rm{\Pi }}}_{a}={\bf{1}}$. For simplicity, we take *θ*_*C*_ to be a half of the maximally entangled state. As described in the introduction, the sender, Alice, performs a joint measurement ${\{{{\rm{\Pi }}}_{a}\}}_{a=1}^{N}$ on *θ*_*C*_ and her share of the resource state. She then communicates the classical outcome $i\in \mathrm{\{1,}\ldots ,N\}$ to Bob, who then traces out all but the *i*-th port which contains the teleported state.

The entanglement fidelity of the protocol for any $d\ge 2$, $N\ge 2$ is given by:

**Theorem 12**. *The fidelity for Port-Based Teleportation is given by* $F=\frac{1}{{d}^{N+2}}{\sum }_{\alpha  \vdash n-2}{({\sum }_{\mu \in \alpha }\sqrt{{d}_{\mu }{m}_{\mu }})}^{2}$, *where sums over* α *and* μ *are taken*, *whenever number of rows in corresponding Young diagrams is not greater than the dimension of the local Hilbert space d*.

**Figure 3:**
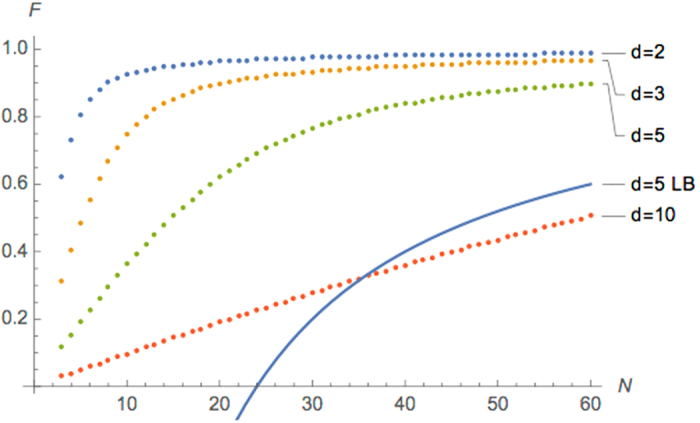
Performance of the deterministic PBT protocol for $d\in \{2,\,3,\,5,\,10\}$. Dotted line denotes explicit values for the entanglement fidelity computed by our algorithm. Solid line denotes the best lower bound for *d* = 5 derived in ref. 10.

From the entanglement fidelity computed in Theorem 12 one can easily obtain the average fidelity using $f=(Fd+\mathrm{1)}/(d+\mathrm{1)}$. Figure 3 shows the performance of the deterministic PBT when Alice wishes to teleport higher-dimensional states.

### Discussion and open questions

We found explicit expressions for the performance of all variations of the PBT in arbitrary dimension with any number of ports. We expect the tools and techniques introduced here to find a number of applications ranging from the study of quantum states with restricted symmetries and calculating properties of the antiferromagnetic systems to problems in the quantum measurement theory.

Our successful approach to studying properties of the port-based teleportation may be replicated for the study of arbitrary systems with partial symmetries. First, one needs to classify the symmetries of the system ($S(n-\mathrm{1)}$ and $S(n-\mathrm{2)}$ in the case of the PBT). Next, to identify and study the structure and the natural representation of the algebra corresponding to the elements with these symmetries (${{\mathscr{A}}}_{n}^{{t}_{n}}(d)$ in the case of the PBT). Finally, to compute various tracial quantities of interest efficiently one needs to adjust the theory PRIRs to account for the symmetries of the system in question.

**Figure 4:**
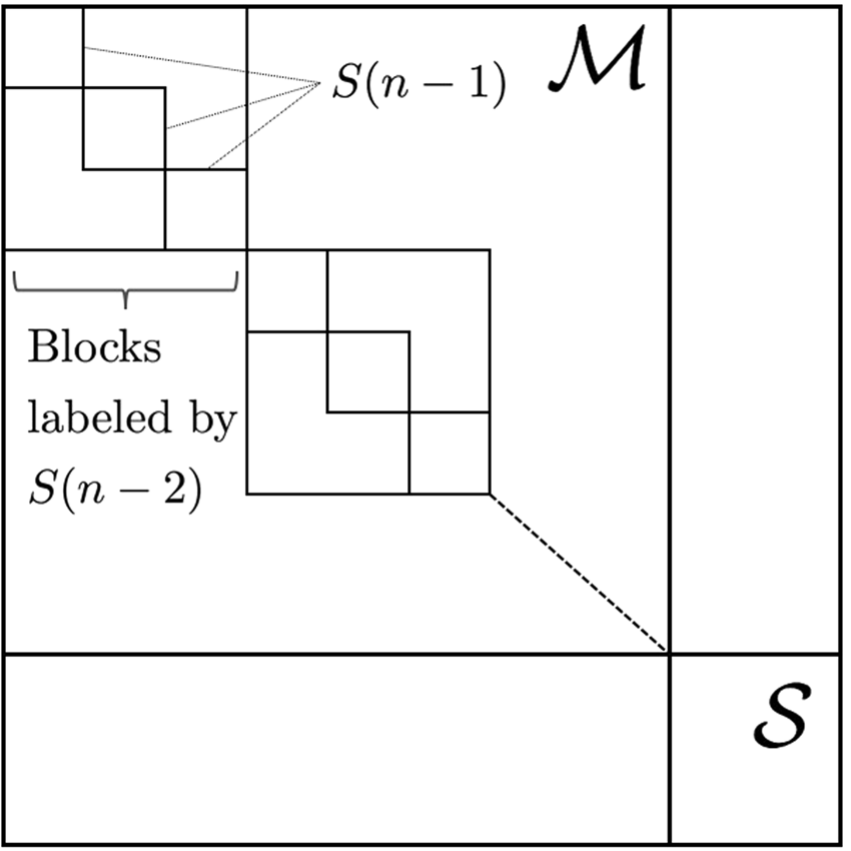
The structure of ${{\mathscr{A}}}_{n}^{{t}_{n}}(d)$. It splits into direct sum of two ideals ${\mathcal M}$ and ${\mathscr{S}}$. The irreps of ${\mathcal M}$ are labelled by the irreps of $S(n-\mathrm{2)}$ and they are strictly connected with the representations of the group $S(n-\mathrm{1)}$ induced from the irreps of $S(n-\mathrm{2)}$ ^18^.

We now mention some open questions. Firstly, how to characterize entanglement content in the resource state after one run of any PBT protocol for *d* > 2. We know that in the qubit case the residual entanglement may be recycled to teleport more states^15^. In addition, when probabilistic PBT fails, Alice can nevertheless make use of the standard teleportation protocol reliably^16^. One might wonder if it is possible to similarly utilize the residual entanglement of the resource state in the qudit case. In particular, to show that such higher-dimensional teleportation scheme works one needs to extend our analysis to the properties of the left ideal ${\mathscr{S}}$ depicted in Fig. 4.

Second, for the probabilistic PBT protocol we have shown that the measurement operators are optimal for a fixed resource state of the form $|{{\rm{\Phi }}}^{+}{\rangle }^{\otimes N}$. We do not know whether this holds for the deterministic PBT in our case. From refs 10, 11, 17 we know that for both the KLM scheme and the PBT protocols teleporting qubits, the resource state and the corresponding measurement differ when optimized simultaneously.

Another important problem is to determine the asymptotic performance of the PBT in arbitrary dimension. This presents a challenging task in particular because the asymptotic representation theory for the regime $d/N\to 0$ is still in its infancy.

## Methods

### Structure of the port-based teleportation operator

In order to quantify the effect of measurement ${\mathscr{X}}$ one has to find the spectral properties of *ρ*. To simplify the analysis, we represent *ρ* in a different form. First, observe that every unnormalised projector ${P}_{C{A}_{a}}^{+}$ can be written as ${P}_{C{A}_{a}}^{+}={V}_{(C{A}_{a})}^{{t}_{C}}$, where ${V}_{(C{A}_{a})}$ denotes a permutation operator between systems *C* and *A*_*a*_, and *t*_*C*_ denotes a transposition with respect to subsystem *C*. Therefore, *ρ* in Eqn. (1) may be written in terms of partially transposed permutation operators:

2 $$\rho =\frac{1}{{d}^{N}}\sum _{a=1}^{N}{V}_{(C{A}_{a})}^{{t}_{C}}\otimes {{\bf{1}}}_{\overline{{A}_{a}}}\mathrm{.}$$

Every element ${V}_{(C{A}_{a})}^{{t}_{C}}\otimes {{\bf{1}}}_{\overline{{A}_{a}}}$ acts as a permutation operator on a full $n=N+1$-particle (we will use *n* − 1 and *N* interchangeably) Hilbert space ${\mathcal H} ={({{\mathbb{C}}}^{d})}^{\otimes n}$. When it is clear from the context, we denote every operator ${V}_{(C{A}_{a})}^{{t}_{C}}\otimes {{\bf{1}}}_{\overline{{A}_{a}}}$ just by ${V}_{(C{A}_{a})}^{{t}_{C}}$. The above form enables us to identify *ρ* as the element of a recently studied algebra of partially transposed permutation operators ${{\mathscr{A}}}_{n}^{{t}_{n}}(d)$ acting in the space ${({{\mathbb{C}}}^{d})}^{\otimes n}$, where $d\in {\mathbb{N}}$ and $d\ge 2$ ^18, 19^. It turns out that ${{\mathscr{A}}}_{n}^{{t}_{n}}(d)$ decomposes into a direct sum of two types of left ideals (A left ideal of an algebra ${\mathscr{A}}$ is a subalgebra ${\mathcal I} \subset {\mathscr{A}}$ such that $ax\in  {\mathcal I}$ whenever $a\in {\mathscr{A}}$ and $x\in  {\mathcal I}$) ${{\mathscr{A}}}_{n}^{{t}_{n}}(d)= {\mathcal M} \oplus {\mathscr{S}}$. To describe the functioning of the PBT protocols it suffices to only consider ${\mathcal M}$. The latter includes the irreducible representations of ${{\mathscr{A}}}_{n}^{{t}_{n}}(d)$ indexed by the irreducible representations of the group $S(n-\mathrm{2)}$ which are strictly connected with the representations of the group $S(n-\mathrm{1)}$ ^18, 19^ (see Fig. 4). To keep the notation consistent with the previous analysis of the algebra ${{\mathscr{A}}}_{n}^{{t}_{n}}(d)$, we consider operator *ρ* without factor $1/{d}^{n-1}$ and we change the numbering of the subsystems rewriting the general form of equation (2) as:

3 $$\eta =\sum _{a=1}^{n-1}{V}^{{t}_{n}}(a,n),$$

where *t*_*n*_ denotes a partial transposition on the *n* ^th^ subsystem, and (*a*, *n*) is a permutation between subsystems *a* and *n*. Here, the subsystem *C* is labelled by *n*. To evaluate the performance of the PBT scheme explicitly, we need to characterize the spectral properties of the above operator given in (3). For $d=2$ and $N\ge 2$ this was done in ref. 10 using the CG coefficients. The constraint for the dimension cannot be improved because the CG formalism does not admit closed-form solutions beyond $d=2$. Our first contribution is the operator decomposition of *η* which leads to a universal decomposition method which works for all $d\ge 2$ and $N\ge 2$.

**Theorem 1**. The operator $\eta ={\sum }_{a=1}^{n-1}{V}^{{t}_{n}}(a,n)$ has the form:

4 $$\eta =\mathop{\oplus }\limits_{\alpha  \vdash n-2}\mathop{\oplus }\limits_{\mu  \vdash n-1\mu \in \alpha }{\eta }_{\mu }(\alpha )=\sum _{\alpha  \vdash n-2}\,\sum _{\mu  \vdash n-1\mu \in \alpha }{P}_{\mu }\sum _{a=1}^{n-1}V(a,n-1){P}_{\alpha }{V}^{{t}_{n}}(n-1,\,n)V(a,n-1),$$

where *α*, *μ* run over Young diagrams whose heights are not greater then the dimension *d* of a single system, $\mu \in \alpha$ denotes a valid Young diagram *μ* obtained from *α* by adding one box in a proper way, ${P}_{\mu },{P}_{\alpha }$ denote Young projectors onto irreps of $S(n-\mathrm{1),}\,S(n-\mathrm{2)}$ labelled by $\mu \, \vdash \,n-\mathrm{1,}\,\alpha \, \vdash \,n-2$ respectively. Each ${\eta }_{\mu }(\alpha )$ is proportional to a projector, i.e. ${\eta }_{\mu }(\alpha )={\gamma }_{\mu }(\alpha ){F}_{\mu }(\alpha )$, ${\gamma }_{\mu }(\alpha )\in {\mathbb{C}}$, ${F}_{\mu }(\alpha )$ is a projector of the dimension ${\rm{\dim }}\,{F}_{\mu }(\alpha )={d}_{\mu }{\tilde{m}}_{\alpha }$, where ${\tilde{m}}_{\alpha }$ is the multiplicity of the irrep of the algebra labelled by *α* in ${{\mathscr{A}}}_{n}^{{t}_{n}}(d)$, and *d*_*μ*_ is the dimension of the irrep of $S(n-\mathrm{1)}$ labelled by *μ*. The projectors ${F}_{\mu }(\alpha )$ satisfy ${F}_{\mu }(\alpha )={M}_{\alpha }{P}_{\mu }$, where *M*_*α*_ is projector including multiplicities onto *α*-th irrep of the algebra ${{\mathscr{A}}}_{n}^{{t}_{n}}(d)$.

**Figure 5:**
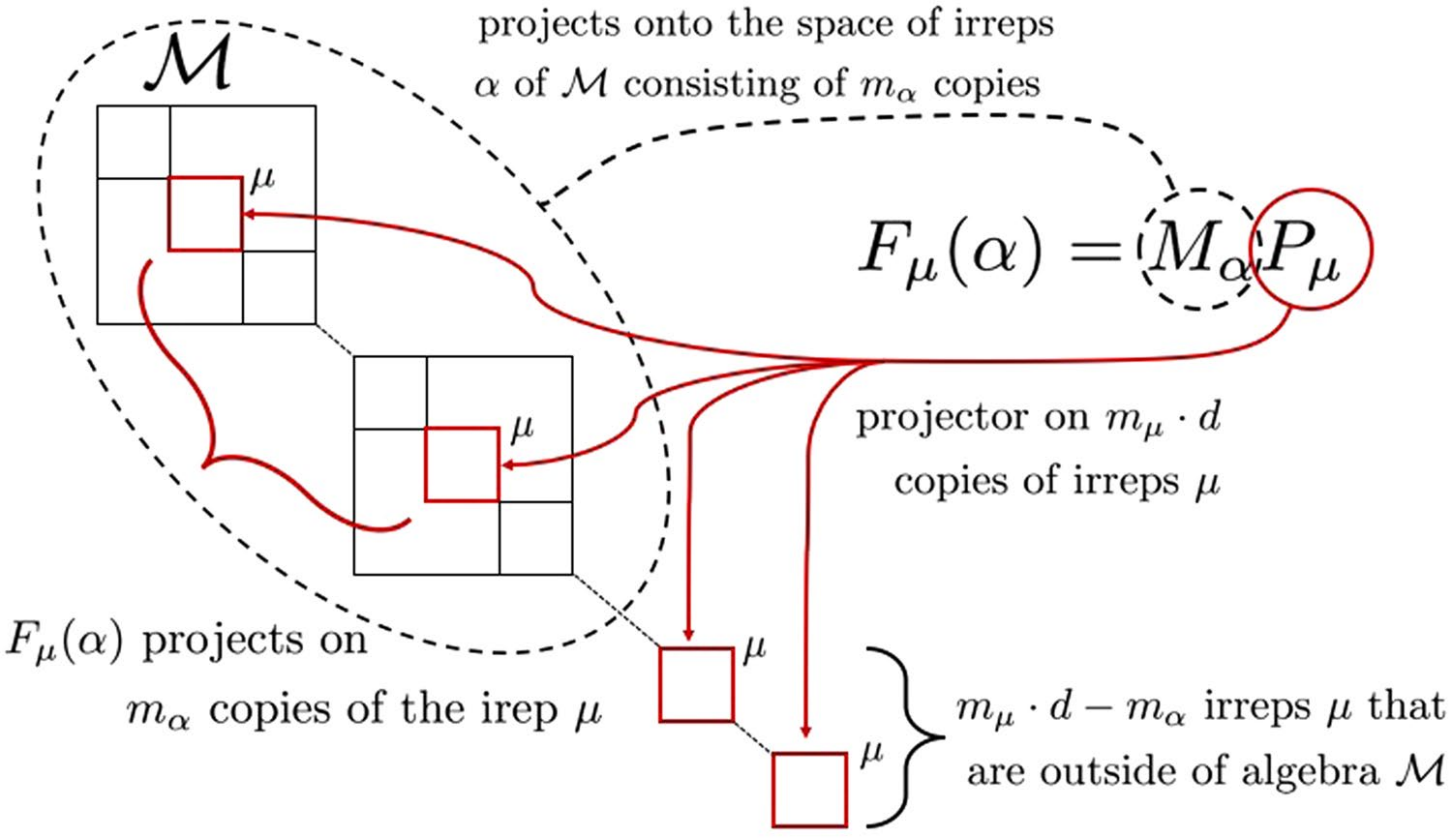
Graphical illustration of the action of the projector ${F}_{\mu }(\alpha )$ in Theorem 1.

The structure and relation of projectors that appear in the theorem are depicted in Fig. 5.

***Proof*** *.* Fix an arbitrary representation of algebra ${{\mathscr{A}}}_{n}^{{t}_{n}}(d)$. Let ${M}_{\alpha }$ be projector (including multiplicities) onto irrep labelled by $\alpha \, \vdash \,n-2$; denote the corresponding subspace $S({M}_{\alpha })$, and set ${P}_{\mu }$ to be a projector onto irrep of $S(n-\mathrm{1)}$ in the same representation (including the multiplicities). Our first goal is to determine the restriction of the operator *η* to the irrep labelled by *α*. We express *η* in terms of operators $\{{v}_{ij}^{ab}(\alpha )\}$, where $1\le a,b\le n-1$ and $1\le i,j\le {d}_{\alpha }$ (see Definition 5 in ref. 19)

5 $${v}_{ij}^{ab}(\alpha )=V(a,n-\mathrm{1)}{E}_{ij}^{\alpha }{V}^{{t}_{n}}(n-\mathrm{1,}n)V(b,n-\mathrm{1),}$$

since they span irrep labelled by *α*. The operators ${\{{E}_{ij}^{\alpha }\}}_{i,j=1}^{{d}_{\alpha }}$ form an operator basis in the irrep $\alpha \, \vdash \,n-2$ of $S(n-\mathrm{2)}$ (see Appendix F for the details). Using (5) we can decompose *η*:

6 $$\begin{array}{rcl}\eta  & = & \sum _{a=1}^{n-1}V(a,n-\mathrm{1)}{V}^{{t}_{n}}(n-\mathrm{1,}\,n)V(a,n-\mathrm{1)}\\  & = & \sum _{\alpha }\sum _{a\mathrm{=1}}^{n-1}V(a,n-\mathrm{1)}{P}_{\alpha }{V}^{{t}_{n}}(n-\mathrm{1,}\,n)V(a,n-\mathrm{1)}\\  & = & \sum _{\alpha }\sum _{i\mathrm{=1}}^{{d}_{\alpha }}\sum _{a\mathrm{=1}}^{n-1}V(a,n-\mathrm{1)}{E}_{ii}^{\alpha }{V}^{{t}_{n}}(n-\mathrm{1,}\,n)V(a,n-\mathrm{1)}\\  & = & \sum _{\alpha }\sum _{i\mathrm{=1}}^{{d}_{\alpha }}\sum _{a\mathrm{=1}}^{n-1}{v}_{ii}^{aa}(\alpha )=\sum _{\alpha }\eta (\alpha ),\end{array}$$

where

7 $$\eta (\alpha )=\sum _{i=1}^{{d}_{\alpha }}{v}_{ii}^{aa}(\alpha )=\sum _{a=1}^{n-1}V(a,n-\mathrm{1)}{P}_{\alpha }{V}^{{t}_{n}}(n-\mathrm{1,}\,n)V(a,n-\mathrm{1).}$$

Thus the support of $\eta (\alpha )$ is precisely the space $S({M}_{\alpha })$ which is invariant under the action of $S(n-\mathrm{1)}$, hence its eigenprojectors are ${F}_{\mu }(\alpha )={M}_{\alpha }{P}_{\mu }$, and this results in the following decomposition:

8 $$\eta (\alpha )=\mathop{\oplus }\limits_{\mu  \vdash n-1\mu \in \alpha }{\gamma }_{\mu }(\alpha ){M}_{\alpha }{P}_{\mu }=\mathop{\oplus }\limits_{\mu \in \alpha }{\eta }_{\mu }(\alpha ),\quad {\gamma }_{\mu }(\alpha )\in {\mathbb{C}},$$

with ${\eta }_{\mu }(\alpha )={P}_{\mu }\eta (\alpha ){P}_{\mu }$. This immediately implies that

9 $${F}_{\mu }(\alpha )={\gamma }_{\mu }^{-1}(\alpha ){P}_{\mu }\eta (\alpha ){P}_{\mu }\mathrm{.}$$

All of the structural properties above are derived solely from the properties of the underlying algebra, and are thus independent of representation. It is known that for any representation $\mathrm{Tr}[{P}_{\mu }{M}_{\alpha }]={d}_{\mu }{\mathop{m}\limits^{ \sim }}_{\alpha }$, where ${\tilde{m}}_{\alpha }$ is the multiplicity of the projector ${M}_{\alpha }$ ^20^.□

To simplify the presentation, we may occasionally switch to the natural representation (for instance when we want to compute the partial trace). A number of fundamental results about the structure of the above algebra was obtained by refs 18 and 19 who in particular showed that ${\tilde{m}}_{\alpha }={m}_{\alpha }$, where *m*_*α*_ is the multiplicity of irrep labelled by *α* in the natural representation of the group *S*(*n* – 2). Keeping all the notation introduced in the previous theorem we now find the formula for the eigenvalues of the operator *η* as well as port-based teleportation operator *ρ*.

**Proposition 2**. [*Extended version*] *The numbers* ${\gamma }_{\mu }(\alpha )$ *given by* *Eqn*. (8) *are the eigenvalues of the operator η given by*

10 $${\gamma }_{\mu }(\alpha )=(n-\mathrm{1)}\frac{{m}_{\mu }{d}_{\alpha }}{{m}_{\alpha }{d}_{\mu }}$$

*or equivalently*:

11 $${\gamma }_{\mu }(\alpha )=d+\frac{1}{2}(n-\mathrm{1)(}n-\mathrm{2)}\frac{{\chi }^{\mu }\mathrm{(12)}}{{d}_{\mu }}-\frac{1}{2}(n-\mathrm{2)(}n-\mathrm{3)}\frac{{\chi }^{\alpha }\mathrm{(12)}}{{d}_{\alpha }}\mathrm{.}$$

*By* ${m}_{\alpha },{m}_{\mu }$ *we denote multiplicities of α*, *μ of S*(*n* – 2) *S* (*n* – 1) *respectively in the natural representation*, *by* ${d}_{\alpha },{d}_{\mu }$ *the respective dimensions*, *and by* ${\chi }^{\mu }\mathrm{(12),}\,{\chi }^{\alpha }\mathrm{(12)}$ *the characters calculated on the transposition* (12) *of the corresponding irreps. By μ we denote Young diagrams obtained from Young diagrams α of* n−2 *boxes by adding a single box in a proper way. We take Young diagrams α,μ whose height is not greater then dimension d of the local Hilbert space*.

***Proof*** *.* From Theorem 1 we know that $\eta ={\sum }_{\mu  \vdash n-1\mu \in \alpha }{\gamma }_{\mu }(\alpha ){F}_{\mu }(\alpha )$, and ${\eta }_{\mu }(\alpha )={\gamma }_{\mu }(\alpha ){F}_{\mu }(\alpha )$. Thus ${\gamma }_{\mu }(\alpha )$ can be expressed as

12 $${\gamma }_{\mu }(\alpha )=\frac{{\rm{Tr}}{\eta }_{\mu }(\alpha )}{{\rm{Tr}}{F}_{\mu }(\alpha )}=\frac{{\rm{Tr}}{\eta }_{\mu }(\alpha )}{{d}_{\mu }{m}_{\alpha }}\mathrm{.}$$

In the last step we need to compute $\mathrm{Tr}{\eta }_{\mu }(\alpha )$. Using the decomposition given in equation (4) and Fact 20 from Appendix C we can simplify:

13 $${\rm{Tr}}{\eta }_{\mu }(\alpha )=(n-\mathrm{1)}{\rm{Tr}}[{P}_{\mu }{P}_{\alpha }{V}^{{t}_{n}}(n-\mathrm{1,}\,n)]={\rm{Tr}}[{P}_{\mu }({P}_{\alpha }\otimes {\bf{1}})]=(n-\mathrm{1)}{m}_{\mu }{d}_{\alpha }\mathrm{.}$$

Plugging (13) into (12) we obtain first statement of the proposition given in equation (10).

To prove (11) we use (12) and (13) express ${\gamma }_{\mu }(\alpha )$ as:

14 $${\gamma }_{\mu }(\alpha )=\frac{n-1}{{d}_{\mu }{m}_{\alpha }}{\rm{Tr}}[{P}_{\mu }({P}_{\alpha }\otimes {\bf{1}})]\mathrm{.}$$

Using Fact 19 from Appendix C to obtain the decomposition of *P*_*μ*_ and simplifying it further we get:

15 $$\begin{array}{rcl}{\gamma }_{\mu }(\alpha ) & = & \frac{n-1}{{m}_{\alpha }(n-\mathrm{1)!}}\sum _{a=1}^{n-1}\sum _{i,j=1}^{{d}_{\mu }}{\phi }_{ij}^{\mu }(a,n-\mathrm{1)}{\rm{Tr}}[V(a,n-\mathrm{1)}{F}_{ij}^{\mu }({P}_{\alpha }\otimes {\bf{1}})]\\  & = & \frac{n-1}{{m}_{\alpha }(n-\mathrm{1)!}}\sum _{a=1}^{n-1}\sum _{i,j=1}^{{d}_{\mu }}{d}^{{\delta }_{a,n-1}}{\phi }_{ij}^{\mu }(a,n-\mathrm{1)}{\rm{Tr}}[{F}_{ij}^{\mu }{P}_{\alpha }],\end{array}$$

where

16 $${F}_{ij}^{\mu }=\sum _{\pi \in S(n-\mathrm{2)}}{\phi }_{ji}^{\mu }({\pi }^{-1})V(\pi \mathrm{).}$$

From Eqn. (67) in Fact 19 in Appendix C and the orthogonality property that ${\rm{Tr}}[{E}_{ij}^{\beta }{P}_{\alpha }]={\sum }_{k}{\rm{Tr}}[{E}_{ij}^{\beta }{E}_{kk}^{\alpha }]=$ ${\rm{Tr}}[{E}_{ij}^{\alpha }]={\delta }_{ij}{m}_{\alpha }$, we write (15) in the PRIR notation defined in Appendix B

17 $$\begin{array}{l}{\gamma }_{\mu }(\alpha )=\frac{1}{{d}_{\alpha }}\sum _{a=1}^{n-1}{d}^{{\delta }_{a,n-1}}(\sum _{{i}_{\alpha }=1}^{{d}_{\alpha }}{({\phi }_{R}^{\mu })}_{{i}_{\alpha }{i}_{\alpha }}^{\alpha \alpha }(a,n-\mathrm{1)})\mathrm{.}\end{array}$$

Finally, using Corollary 18 from Appendix B we obtain the second statement of the proposition.

Thus, the eigenvalues of the PBT operator *ρ* given in Eqn. (1) are the rescaled version of the above:

18 $${\lambda }_{\mu }(\alpha )=(1/{d}^{N}){\gamma }_{\mu }(\alpha )=(N/{d}^{N})\frac{{m}_{\mu }{d}_{\alpha }}{{m}_{\alpha }{d}_{\mu }}\mathrm{.}$$

### Probabilistic version of the protocol

In the following two subsections we find maximal average success probability when a resource state is the maximally entangled state and then show how to find optimal resource state and POVMs simultaneously.

To prove the optimality in the above cases, we formulate the question as a semidefinite program. We prove the main theorems by presenting feasible solutions for a primal and a dual semidefinite problem. By establishing the solution to a primal problem we obtain an achievable lower bound for the success probability; the corresponding solution to the dual yields the upper bound. Observing that the respective bounds coincide we arrive at the optimal probability of success P_opt_.

#### Maximally entangled state as a resource state

From ref. 12 it follows that the optimal POVMs for the probabilistic PBT coincides with the ones for distinguishing the set of states ${\{(1/N;{\rho }_{a})\}}_{a=1}^{N}$. We thus look for a set of POVMs ${\{{{\rm{\Pi }}}_{a}={P}_{a,n}^{+}\otimes {\Theta }_{\overline{a}}\}}_{a=1}^{N}$ which would maximize the average success probability

19 $${p}^{\ast }=\frac{1}{{d}^{N+1}}\sum _{a=1}^{N}{\rm{Tr}}{\Pi }_{a}=\frac{1}{{d}^{N+1}}\sum _{a=1}^{N}{\rm{Tr}}{\Theta }_{\overline{a}}$$

subject to:

20 $$\,\mathrm{(1)}\,{\Theta }_{\overline{a}}\ge \mathrm{0,}\quad \,\mathrm{(2)}\,\sum _{a=1}^{N}{P}_{a,n}^{+}\otimes {\Theta }_{\overline{a}}\le {{\bf{1}}}_{AB}\quad a=\mathrm{1,}\,\mathrm{2,}\ldots ,N\mathrm{.}$$

Since our resource state is maximally entangled, the RHS of the second constraint in (20) reduces to identity on *AB* (see ref. 10). Our main contribution here is the explicit expression for the probability of success of PBT:

**Theorem 3**. *The maximal average success probability in the probabilistic PBT with a resource state consisting of maximally entangled pairs is given by* ${p}_{opt}=\frac{1}{{d}^{N}}{\sum }_{\alpha }{m}_{\alpha }^{2}{\min }_{\mu \in \alpha }\frac{{d}_{\mu }}{{m}_{\mu }}$, *where μ denotes Young diagram obtained from* $\alpha  \vdash n-2$ *by adding a single box in a proper way and* ${\gamma }_{\mu }(\alpha )$ *is given in Proposition* 2.

**Lemma 4**. The primal is feasible with ${p}^{\ast }=\frac{1}{{d}^{N}}{\sum }_{\alpha }{{\rm{\min }}}_{\mu \in \alpha }\frac{{m}_{\alpha }{d}_{\alpha }}{{\gamma }_{\mu }(\alpha )}\le {p}_{opt}$.

The proof is located in Appendix G.1. The dual problem is to minimize

21 $${p}^{\ast }=\frac{1}{{d}^{N+1}}\mathrm{Tr}{\rm{\Omega }}$$

subject to:

22 $$\quad \mathrm{(1)}\,{\rm{\Omega }}\ge \mathrm{0,}\quad \mathrm{(2)}\,{{\rm{Tr}}}_{a,n}[{P}_{a,n}^{+}{\rm{\Omega }}]\ge {\bf{1}},$$

where $a=\mathrm{1,}\ldots ,n-1$, Ω acts on *n* systems the identity **1** is defined on *n* − 2 systems, and ${P}_{a,n}^{+}$ is projector onto maximally entangled state between respective subsystems. We choose the operator Ω by a linear combination of the projectors ${F}_{\mu }(\alpha )$ defined in Theorem 1

23 $${\rm{\Omega }}=\sum _{\alpha }{x}_{{\mu }^{\ast }}(\alpha ){F}_{{\mu }^{\ast }}(\alpha ),\quad {x}_{{\mu }^{\ast }}(\alpha )\ge \mathrm{0,}$$

where ${\mu }^{\ast }\, \vdash \,n-1$ denotes the Young diagram obtained from $\alpha \, \vdash \,n-2$ by adding one box in a proper way in such a way that ${\gamma }_{{\mu }^{\ast }}(\alpha )$ is possible maximal. From the definition of the constraints (21) and the symmetries of the projectors ${F}_{\mu }(\alpha )$ we need the following fact

**Fact 5**. *Let* ${F}_{\mu }(\alpha )$ *be the operators given in Theorem 1, and let* ${V}^{{t}_{n}}(n-\mathrm{1,}\,n)$ *be a permutation operator acting between* $(n-\mathrm{1)}$ *-th and n-th subsystems partially transposed with respect to n-th subsystem, then* ${{\rm{Tr}}}_{n-\mathrm{1,}n}[{V}^{{t}_{n}}(n-\mathrm{1,}\,n){F}_{\mu }(\alpha )]$ $=\frac{{m}_{\mu }}{{m}_{\alpha }}{P}_{\alpha }$ *, where numbers* ${m}_{\alpha },{m}_{\mu }$ *are the multiplicities of the respective irreps and* ${P}_{\alpha }$ *is the Young projector onto a irreducible subspace labelled by the partition* $\alpha  \vdash n-2$.

The proof of Fact 5 an the lemma below are located in Apendix G.2 and G.3 respectively.

**Lemma 6**. *The dual is feasible with* ${p}^{\ast }=\frac{1}{{d}^{N}}{\sum }_{\alpha }{m}_{\alpha }^{2}\frac{{d}_{{\mu }^{\ast }}}{{m}_{{\mu }^{\ast }}}\ge {p}_{opt}$.

Combining Lemma 4 with Lemma 6 we formulate the following proposition:

**Proposition 7**. *From* ${p}^{\ast }={p}^{\ast }$ *we conclude that* ${\Theta }_{\overline{a}}=d{\sum }_{\alpha }\frac{1}{{\gamma }_{{\mu }^{\ast }}(\alpha )}{P}_{\alpha }=d{\sum }_{\alpha }{P}_{\alpha }{{\rm{\min }}}_{\mu \in \alpha }\frac{1}{{\gamma }_{\mu }(\alpha )}$ *for* $a=\mathrm{1,}\,\mathrm{2,}\ldots ,N$ *are the optimal POVMs for the maximally entangled state as a resource state*.

#### Optimisation over a resource state

We now turn to the case when both the optimal POVMs and a resource state are optimized simultaneously. We thus look for a set of POVMs ${\{{{\rm{\Pi }}}_{a}={P}_{a,n}^{+}\otimes {{\rm{\Theta }}}_{\overline{a}}\}}_{a=1}^{N}$ which would maximize the average success probability

24 $${p}^{\ast }=\frac{1}{{d}^{N+1}}\sum _{a=1}^{N}{\mathrm{Tr}{\rm{\Pi }}}_{a}=\frac{1}{{d}^{N+1}}\sum _{a=1}^{N}{\mathrm{Tr}{\rm{\Theta }}}_{\overline{a}}$$

subject to

25 $$\,\mathrm{(1)}\,{{\rm{\Theta }}}_{\overline{a}}\ge \mathrm{0,}\quad \mathrm{(2)}\,\,\sum _{a=1}^{N}{P}_{a,n}^{+}\otimes {{\rm{\Theta }}}_{\overline{a}}\le {X}_{A}\otimes {{\bf{1}}}_{B}\quad a=\mathrm{1,}\,\mathrm{2,}\ldots ,N\mathrm{.}$$

In the above ${X}_{A}={O}_{A}^{\dagger }{O}_{A}\ge 0$, where *O*_A_ is an operation applied by Alice on her half of the resource state satisfying ${\rm{Tr}}{X}_{A}={d}^{N}$ (see ref. 10). Using our formalism, we derive the optimal state for the probabilistic PBT:

**Theorem 8**. *The optimal state in the probabilistic PBT is given by*, $|{\zeta }_{opt}\rangle =({O}_{A}\otimes {\bf{1}})|{\psi }^{+}{\rangle }^{\otimes N}$ *, where* ${O}_{A}={X}_{A}^{1/2}$ *with*

26 $${X}_{A}=\sum _{\mu }{c}_{\mu }{P}_{\mu }\quad {with}\quad {c}_{\mu }=\frac{{d}^{N}g(N){m}_{\mu }}{{d}_{\mu }},$$

*where* $g(N)=1/{\sum }_{\nu }{m}_{\nu }^{2}$, *and ν labels irreps of S(n−1)*. *Operators* ${P}_{\mu }$ *are Young projectors onto irreps of S(n−1). The optimal set of POVMs in the probabilistic PBT are given by* ${\{{{\rm{\Pi }}}_{a}={P}_{a,n}^{+}\otimes {{\rm{\Theta }}}_{\overline{a}}\}}_{a=1}^{N}$, *where*

27 $$\forall \,a=\mathrm{1,}\ldots ,N\quad {\Theta }_{\overline{a}}=\sum _{\alpha }u(\alpha ){P}_{\alpha ,\overline{a}},\quad {\rm{with}}\quad u(\alpha )=\frac{{d}^{N+1}g(N){m}_{\alpha }}{N{d}_{\alpha }}\mathrm{.}$$

*Above sum runs over all allowed irreps of* $S(n-\mathrm{2)}$, $g(N)=1/{\sum }_{\nu }{m}_{\nu }^{2}$ *for all* $\nu  \vdash n-1$. *By* ${P}_{\alpha ,\overline{a}}$ *we denote Young projectors onto irreps of* $S(n-\mathrm{2)}$ *defined on every subsystem except n-th and a-th. The corresponding optimal probability is of the form* ${p}_{opt}=1-\frac{{d}^{2}-1}{N+{d}^{2}-1}$, *where N is the number of ports, and d is the dimension of the local Hilbert space*.

We prove the above theorem by presenting feasible solutions to a primal (Lemma 9), auxiliary lemma (Lemma 10) and dual semidefinite problem (Lemma 11). Proofs can be found in Appendix G.4, G.5 and G.6 respectively. Defining again a feasible value of the primal problem as in (19), but with respect to constraints (25) we have.

**Lemma 9**. *The primal is feasible with* $p\ast =1-\frac{{d}^{2}-1}{N+{d}^{2}-1}$ *, where N is the number of ports, and d is the dimension of the local Hilbert space*.

The dual problem is of minimizing ${p}^{\ast }={d}^{N}b$, where $b\in {{\mathbb{R}}}_{+}$ subject to

28 $$\mathrm{(1)}\,{\rm{\Omega }}\ge \mathrm{0,}\quad \,\mathrm{(2)}\,{{\rm{Tr}}}_{a,n}[{P}_{a,n}^{+}{\rm{\Omega }}]\ge {{\bf{1}}}_{1\ldots n-2},\quad \,\mathrm{(3)}\,b{{\bf{1}}}_{1\ldots n-1}-\frac{1}{{d}^{N+1}}{{\rm{Tr}}}_{a}{\rm{\Omega }}\ge \mathrm{0,}$$

where $a=\mathrm{1,}\ldots ,n-1$, Ω acts on *n* systems, identities ${{\bf{1}}}_{1\ldots n-2},{{\bf{1}}}_{1\ldots n-1}$ are defined on $n-2$ and $n-1$ systems respectively, and ${P}_{a,n}^{+}$ is a projector onto maximally entangled state between respective subsystems. As in the case of the maximally entangled state we assume the general form of Ω to be given as a linear combination of the projectors ${F}_{\mu }(\alpha )$ defined in Theorem 1

29 $${\rm{\Omega }}=\sum _{\alpha }\sum _{\mu \in \alpha }{x}_{\mu }(\alpha ){F}_{\mu }(\alpha ),\quad {x}_{\mu }(\alpha )\ge \mathrm{0,}$$

where $\mu \, \vdash \,n-1$ denotes the Young diagram obtained from the Young diagram $\alpha  \vdash n-2$ by adding one box in a proper way. From the definition of the constraints (28) and the symmetries of the projectors ${F}_{\mu }(\alpha )$ we calculate its partial trace in terms of ${P}_{\mu }$:

**Lemma 10**. *Let* ${F}_{\mu }(\alpha )$ *be the operators given in Theorem 1, then* ${{\rm{Tr}}}_{n}{F}_{\mu }(\alpha )=\frac{{m}_{\alpha }}{{m}_{\mu }}{P}_{\mu }$ *, where numbers* ${m}_{\alpha },{m}_{\mu }$ *are multiplicities of the respective irreps and* ${P}_{\mu }$ *is the Young projector onto irreducible subspace labelled by the partition* $\mu  \vdash n-1$.

Having Lemma 10 we are ready to formulate the following:

**Lemma 11**. *The dual is feasible with* ${p}^{\ast }=1-\frac{{d}^{2}-1}{N+{d}^{2}-1}$ *, where N is the number of ports, and d is the dimension of the local Hilbert space*.

By combining Lemma 9 and Lemma 11 we find that ${p}^{\ast }={p}^{\ast }$, so we conclude that ${\Theta }_{\overline{a}}$ for $a=\mathrm{1,}\,\mathrm{2,}\ldots ,N$ given in (108) are the optimal POVMs with the optimal state given by (109), concluding the proof of Theorem 8.

One important consequence of Theorem 8 is that the optimal probability of success in probabilistic performance of port-based teleportation is given only in terms of ‘global’ parameters such as the number of ports *N* and the dimension of a local Hilbert space *d*, $p\equiv p(d,N)$. Thus, for any fixed dimension *d* we get ${\mathrm{lim}}_{N\to \infty }p(d,N)=1$, which shows that in the asymptotic limit our protocol achieves a unit success probability. Moreover, for fixed number of ports *N* we have ${\mathrm{lim}}_{d\to \infty }p(d,N)=0$ as we expected. Since the probability of success given in in Theorem 8 is optimal and upper bounds the probability of success in Theorem 3 in the case of maximally entangled state for any value of *d* and *N*.

### Deterministic version of the protocol

The deterministic version of the PBT is described by *N* POVM elements ${\{{{\rm{\Pi }}}_{a}\}}_{a=1}^{N}$ with each Πa = ρ −1/2ρ aρ −1/2+ΔΠa = ρ −1/2ρ aρ −1/2+Δ, where the term ${\rm{\Delta }}=\mathrm{(1}/N)({\bf{1}}-{\sum }_{a=1}^{N}{{\rm{\Pi }}}_{a})$ with ${\rm{Tr}}{\rho }_{a}{\rm{\Delta }}=0$ is required to ensure that ${\sum }_{a=1}^{N}{{\rm{\Pi }}}_{a}={\bf{1}}$. For simplicity, we take ${\theta }_{C}$ to be a half of the maximally entangled state. As described in the introduction, the sender, Alice, performs a joint measurement ${\{{{\rm{\Pi }}}_{a}\}}_{a=1}^{N}$ on ${\theta }_{C}$ and her share of the resource state. The entanglement fidelity of the protocol for any $d\ge 2$, N ≥ 2d ≥ 2, N ≥ 2 is given by:

**Theorem 12**. *The fidelity for Port-Based Teleportation is given by the following formula* $F=\frac{1}{{d}^{N+2}}{\sum }_{\alpha  \vdash n-2}$ ${({\sum }_{\mu \in \alpha }\sqrt{{d}_{\mu }{m}_{\mu }})}^{2}$ *, where sums over α and μ are taken, whenever number of rows in corresponding Young diagrams is not greater than the dimension of the local Hilbert space d*.

***Proof***. From ref. 10 we know that the fidelity in PBT is given by

30 $$F=\frac{1}{{d}^{2}}\sum _{a=1}^{N}{\rm{Tr}}[{\rho }_{a}{\rho }^{-\mathrm{1/2}}{\rho }_{a}{\rho }^{-\mathrm{1/2}}]=\frac{N}{{d}^{N+2}}{\rm{Tr}}[{V}^{{t}_{n}}(n-\mathrm{1,}\,n){\eta }^{-\mathrm{1/2}}{V}^{{t}_{n}}(n-\mathrm{1,}\,n){\eta }^{-\mathrm{1/2}}],$$

where the right-hand side is recast using our notation. We use fact that values ${\rm{Tr}}[{\rho }_{a}{\rho }^{-1/2}{\rho }_{a}{\rho }^{-1/2}]$ do not depend on *a*, so it suffices to evaluate (30) for the simplest case, when *a* = *N*, then ${V}^{{t}_{n}}(n-\mathrm{1,}n)={\bf{1}}\otimes {P}_{n-\mathrm{1,}n}^{+}\equiv {\bf{1}}\otimes {P}_{+}$. In this proof *P*_+_ is an unnormalized projector onto a maximally entangled state between $(n-\mathrm{1)}$-th and *n*-th subsystem - we will use notation *P*_+_ and ${V}^{{t}_{n}}(n-\mathrm{1,}\,n)$ interchangeably. Using Theorem 1 we get:

31 $$\begin{array}{ccc}F & = & \frac{N}{{d}^{N+2}}{\rm{T}}{\rm{r}}[({\bf{1}}\otimes {P}_{+}){\eta }^{-1/2}({\bf{1}}\otimes {P}_{+}){\eta }^{-1/2}]\\  & = & \frac{N}{{d}^{N+2}}\sum _{\alpha ,\alpha {\rm{^{\prime} }}}\,\sum _{\mu \in \alpha \mu {\rm{^{\prime} }}\in \alpha {\rm{^{\prime} }}}\frac{{\zeta }_{\mu ,\mu {\rm{^{\prime} }}}(\alpha ,\alpha {\rm{^{\prime} }})}{\sqrt{{\gamma }_{\mu }(\alpha )}\sqrt{{\gamma }_{\mu {\rm{^{\prime} }}}(\alpha {\rm{^{\prime} }})}},\end{array}$$

where ${\zeta }_{\mu ,\mu ^{\prime} }(\alpha ,\alpha ^{\prime} )={\rm{Tr}}\lfloor {V}^{{t}_{n}}(n-\mathrm{1,}\,n){F}_{\mu }(\alpha ){V}^{{t}_{n}}(n-\mathrm{1,}\,n){F}_{\mu ^{\prime} }(\alpha ^{\prime} )\rfloor$. Using Fact 13 from Appendix A we rewrite the trace as follows:

32 $${\zeta }_{\mu ,\mu ^{\prime} }(\alpha ,\alpha ^{\prime} )={\rm{Tr}}\,[{V}^{{t}_{n}}(n-\mathrm{1,}\,n){P}_{\mu }{M}_{\alpha }{V}^{{t}_{n}}(n-\mathrm{1,}\,n){P}_{\mu ^{\prime} }{M}_{\alpha ^{\prime} }]$$

33 $$={\rm{Tr}}\,[{P}_{\alpha ^{\prime} }{V}^{{t}_{n}}(n-\mathrm{1,}\,n){P}_{\mu }{P}_{\alpha }{V}^{{t}_{n}}(n-\mathrm{1,}\,n){P}_{\mu ^{\prime} }]\mathrm{.}$$

After further simplification (see Appendix D for details), we further reduce the expression inside of the trace to get:

34 $${\zeta }_{\mu ,\mu ^{\prime} }(\alpha ,\alpha ^{\prime} )=\sum _{a,b\mathrm{=1}}^{n-1}\sum _{i,j\mathrm{=1}}^{{d}_{\mu }}\frac{{d}_{\mu }{d}_{\mu ^{\prime} }{d}^{{\delta }_{a,n-1}}{d}^{{\delta }_{b,n-1}}}{{[(n-\mathrm{1)!}]}^{2}}{\phi }_{ij}^{\mu }(a,n-\mathrm{1)}{\phi }_{kl}^{\mu ^{\prime} }(b,n-\mathrm{1)}{\rm{Tr}}[({P}_{\alpha }{F}_{ij}^{\mu })({P}_{\alpha ^{\prime} }{F}_{kl}^{\mu ^{\prime} })],$$

where ${F}_{ij}^{\mu }$ are defined in Fact 19 of Appendix C. The operators ${F}_{ij}^{\mu },{F}_{kl}^{\mu ^{\prime} }$ can be expressed as direct sum of operators ${E}_{st}^{\beta }$ as in Eqn. (67) of Appendix C, where $\beta  \vdash n-2$, so

35 $${\rm{Tr}}[({P}_{\alpha }{F}_{ij}^{\mu })({P}_{\alpha ^{\prime} }{F}_{kl}^{\mu ^{\prime} })]=\frac{{[(n-\mathrm{2)!}]}^{2}}{{d}_{\alpha }^{2}}{m}_{\alpha }{\delta }_{\alpha \alpha ^{\prime} }{\delta }_{li}{\delta }_{jk}\mathrm{.}$$

Substituting (35) into (34) and collecting the terms we obtain the following equation:

36 $$F=\frac{1}{N{d}^{N+2}}\sum _{\alpha }\frac{{m}_{\alpha }}{{d}_{\alpha }^{2}}\sum _{\mu \in \alpha \mu {\rm{^{\prime} }}\in \alpha {\rm{^{\prime} }}}\frac{{d}_{\mu }{d}_{\mu {\rm{^{\prime} }}}}{\sqrt{{\gamma }_{\mu }(\alpha ){\gamma }_{\mu {\rm{^{\prime} }}}(\alpha )}}{g}_{\mu ,\mu {\rm{^{\prime} }}}(\alpha ),$$

where the term ${g}_{\mu ,\mu ^{\prime} }(\alpha )={\sum }_{a,b=1}^{n-1}{d}^{{\delta }_{a,n-1}}{d}^{{\delta }_{b,n-1}}{\sum }_{{i}_{\alpha },{j}_{\alpha }=1}^{{d}_{\alpha }}{({\varphi }_{R}^{\mu })}_{{i}_{\alpha }{j}_{\alpha }}^{\alpha \alpha }(a,n-\mathrm{1)(}{\varphi }_{R}^{\mu ^{\prime} }{)}_{{j}_{\alpha }{i}_{\alpha }}^{\alpha \alpha }(b,n-\mathrm{1)}$, evaluated in Appendix D.2, yields the following tractable expression for the fidelity:

37 $$F=\frac{1}{N{d}^{N+2}}\sum _{\alpha }\frac{{m}_{\alpha }}{{d}_{\alpha }}\sum _{\mu \in \alpha \mu {\rm{^{\prime} }}\in \alpha {\rm{^{\prime} }}}{d}_{\mu }{d}_{\mu {\rm{^{\prime} }}}\sqrt{{\gamma }_{\mu }(\alpha ){\gamma }_{\mu {\rm{^{\prime} }}}(\alpha )}.$$

We get the final result by substituting the expression for the eigenvalues ${\gamma }_{\mu }(\alpha ),{\gamma }_{\mu ^{\prime} }(\alpha )$ from Eqn. (10) of Theorem 2.

## Electronic supplementary material

Supplementary Information
