# Supplementary material for: Port-based teleportation in arbitrary dimension
Source: Sci Rep. 2017 Sep 7;7:10871. doi: 10.1038/s41598-017-10051-4 (PMC5589940; doi:10.1038/s41598-017-10051-4)
Supplement: Supplementary file 1 — Supplementary Information [file 41598_2017_10051_MOESM1_ESM.pdf]

Port-based teleportation in arbitrary dimension

Supplementary Information

Michał Studziński, Sergii Strelchuk, Marek Mozrzymas, Michał Horodecki

A Auxilliary fact about operator $V^{t_n}(n-1,n)$

Fact 13. *Let M_α be projector including multiplicities onto α-th irrep of the algebra $\mathscr{A}_n^{t_n}(d)$, let P_α, P_β where $\alpha,\beta \vdash n-2$ be a Young projectors, and let $V^{t_n}(n-1,n)$ be a permutation operator acting between $(n-1)$-th and n-th subsystems partially transposed with respect to n-th subsystem, then*

$$M_\alpha V^{t_n}(n-1,n) = P_\alpha V^{t_n}(n-1,n), \tag{38}$$

Proof. The proof is based on the results presented in[19]. Namely we know that operators $V^{t_n}(\sigma)$, where $\sigma \in S(n)$ can be decomposed in every irrep labelled by α in operator basis $\{v_{ij}^{ab}(\alpha)\}$, where $1 \leq a,b \leq n-1$ and $1 \leq i,j \leq d_\alpha$. In particular, when $\sigma = (n-1,n)$ we have:

$$V^{t_n}(n-1,n) = \sum_{\alpha \vdash n-2} \sum_{i,j=1}^{d_\alpha} \varphi_{ij}^{\alpha}(\mathrm{e}) v_{ij}^{n-1,n-1}(\alpha), \tag{39}$$

where e denotes the identity element of $S(n-2)$. We see that $\sum_{i,j=1}^{d_\alpha} \varphi_{ij}^{\alpha}(\mathrm{e}) v_{ij}^{n-1,n-1}(\alpha) = M_\alpha V^{t_n}(n-1,n)$ is a restriction of $V^{t_n}(\sigma)$ to irrep labelled by α, so rewriting equation (39)

$$M_\alpha V^{t_n}(n-1,n) = \sum_{i,j=1}^{d_\alpha} \varphi_{ij}^{\alpha}(\mathrm{e}) v_{ij}^{n-1,n-1}(\alpha) = \sum_{i=1}^{d_\alpha} v_{ii}^{n-1,n-1}(\alpha), \tag{40}$$

since $\varphi_{ij}^{\alpha}(\mathrm{e}) = \delta_{ij}$. Using equation (5) from Section 2.4 we write:

$$\sum_{i=1}^{d_\alpha} v_{ii}^{n-1,n-1}(\alpha) = \sum_{i=1}^{d_\alpha} E_{ii}^{\alpha} V^{t_n}(n-1,n) = P_\alpha V^{t_n}(n-1,n), \tag{41}$$

since $P_\alpha = \sum_{i=1}^{d_\alpha} E_{ii}^{\alpha}$. This finishes the proof. □

B Partially reduced irreducible representations (PRIR)

In this section we derive some properties of the PRIR. This concept plays a crucial role in the simplification of the representation of the algebra $\mathscr{A}_n^{t_n}(d)$.

Let us consider an arbitrary unitary irrep ϕ^μ of $S(n)$. It can be always unitarily transformed to PRIR ϕ_R^μ such that

$$\forall \pi \in S(n-1) \quad \phi_R^\mu(\pi) = \bigoplus_{\alpha \in \mu} \varphi^\alpha(\pi), \tag{42}$$

where φ^α are irreps of $S(n-1)$. By $\alpha \in \mu$ we denote Young diagrams α which can be obtained from μ by removing one box in the proper way. We see that the restriction of the irrep ϕ^μ of $S(n)$ to the subgroup $S(n-1)$ has a block-diagonal form of completely reduced representation, which in matrix notation takes the form

$$\forall \pi \in S(n-1) \quad \phi_R^\mu(\pi) = \left(\delta^{\alpha\beta} \varphi_{i_\alpha j_\alpha}^{\alpha}\right). \tag{43}$$

The block structure of this reduced representation allows us to introduce such a block indexation for PRIR ϕ_R^μ of $S(n)$, which gives

$$\forall \sigma \in S(n) \quad \phi_R^\mu(\sigma) = \left(\phi_{i_\alpha j_\beta}^{\alpha\beta}(\sigma)\right), \tag{44}$$

where the matrices on the diagonal $(\phi^\mu_R)^{\alpha\alpha}(\sigma) = \left(\phi^{\alpha\alpha}_{i_\alpha j_\alpha}(\sigma)\right)$ are of dimension of corresponding irrep φ^α of $S(n-1)$. The off diagonal blocks need not to be square. The matrices $(\phi^\mu_R)^{\alpha\alpha}(\sigma) = \left(\phi^{\alpha\alpha}_{i_\alpha j_\alpha}(\sigma)\right)$ on the diagonal of the matrix $\phi^\mu_R(\sigma)$ have the following important properties:

Proposition 14. *Let $(\phi^\mu_R)^{\alpha\alpha}(\sigma) = \left(\phi^{\alpha\alpha}_{i_\alpha j_\alpha}(\sigma)\right)$ be the matrices on the diagonal of the PRIR matrix $\phi^\mu_R(\sigma)$, then*

$$\forall \alpha \in \mu \quad \varphi^\alpha(\pi)\left((\phi^\mu_R)^{\alpha\alpha}(an)\right)\varphi^\alpha(\pi^{-1}) = \left((\phi^\mu_R)^{\alpha\alpha}(\pi(a)n)\right), \tag{45}$$

and from this it follows

$$\forall \alpha \in \mu \quad \forall \pi \in S(n-1) \quad \forall a = 1,\dots,n-1 \qquad \mathrm{Tr}\left[(\phi^\mu_R)^{\alpha\alpha}(an)\right] = \mathrm{Tr}\left[(\phi^\mu_R)^{\alpha\alpha}(\pi(a)n)\right], \tag{46}$$

so the trace is constant on the transpositions which naturally indexed the coset $S(n)/S(n-1)$.

Proof. From the composition rule in $S(n)$ we have

$$\forall \pi \in S(n-1) \quad \forall a = 1,\dots,n-1 \qquad \pi\circ(an)\circ\pi^{-1} = (\pi(a)n), \tag{47}$$

which implies

$$\forall \pi \in S(n-1) \quad \forall a = 1,\dots,n-1 \qquad \phi^\mu_R(\pi)\phi^\mu_R(an)\phi^\mu_R(\pi^{-1}) = \phi^\mu_R(\pi(a)n), \tag{48}$$

where the matrices $\phi^\mu_R(\pi)$, $\phi^\mu_R(\pi^{-1})$ are block-diagonal (see expression (43)). From multiplication rule of block diagonal matrices we get that the equation (48) in the irrep ϕ^μ_R yields the following equations for its diagonal blocks determined by the irrep's φ^α of $S(n-1)$

$$\forall \alpha \in \mu \quad \varphi^\alpha(\pi)\left((\phi^\mu_R)^{\alpha\alpha}(an)\right)\varphi^\alpha(\pi^{-1}) = \left((\phi^\mu_R)^{\alpha\alpha}(\pi(a)n)\right). \tag{49}$$

Taking the trace, in $\mathbb{M}(d_\alpha,\mathbb{C})$, on this equation we get the second statement of the Proposition. □

Further we have the following sum rules

Proposition 15. *The PRIR ϕ^μ_R of $S(n)$ satisfies the following sum rules*

$$\sum_{a=1}^{n-1}(\phi^\mu_R)(an) = \frac{n(n-1)}{2}\frac{\chi^\mu(12)}{d_\mu}\mathbf{1}_{\phi^\mu} - \bigoplus_{\alpha\in\mu}\frac{(n-1)(n-2)}{2}\frac{\chi^\alpha(12)}{d_\alpha}\mathbf{1}_{\varphi^\alpha}, \tag{50}$$

which implies that for the diagonal blocks we have

$$\forall \alpha \in \mu \qquad \sum_{a=1}^{n-1}(\phi^\mu_R)^{\alpha\alpha}(an) = \left[\frac{n(n-1)}{2}\frac{\chi^\mu(12)}{d_\mu} - \frac{(n-1)(n-2)}{2}\frac{\chi^\alpha(12)}{d_\alpha}\right]\mathbf{1}_{\varphi^\alpha}. \tag{51}$$

Proof. The starting point is the classical equation

$$\sum_{(ab)\in S(n)}(\phi^\mu_R)(ab) = \frac{n(n-1)}{2}\frac{\chi^\mu(12)}{d_\mu}\mathbf{1}_{\phi^\mu}, \tag{52}$$

which holds for any irrep of $S(n)$. We rewrite *LHS* of equation (52) separating the terms in $S(n-1)$

$$\begin{aligned}\sum_{(ab)\in S(n)}(\phi^\mu_R)(ab) &= \sum_{a=1}^{n-1}(\phi^\mu_R)(an) + \sum_{(cd)\in S(n-1)}(\phi^\mu_R)(cd)\\ &= \sum_{a=1}^{n-1}(\phi^\mu_R)(an) + \bigoplus_{\alpha\in\mu}\sum_{(cd)\in S(n-1)}\varphi^\alpha(cd).\end{aligned} \tag{53}$$

Now we use one more equation (52) to each irrep φ^α in the direct sum of *RHS* in equation (53) we get

$$\sum_{(ab)\in S(n)}(\phi_R^\mu)(ab)=\sum_{a=1}^{n-1}(\phi_R^\mu)(an)+\bigoplus_{\alpha\in\mu}\frac{(n-1)(n-2)}{2}\frac{\chi^\alpha(12)}{d_\alpha}\mathbf{1}_{\varphi^\alpha}. \tag{54}$$

This equation together with expression (52) gives the first statement of the proposition. □

Remark 16. *Equation (50) in Proposition 15 may be written in a more explicit form as follows:*

$$\forall\alpha\in\mu \qquad \sum_{a=1}^{n-1}(\phi_R^\mu)^{\alpha\alpha}_{i_\alpha j_\alpha}(an)=\left[\frac{n(n-1)}{2}\frac{\chi^\mu(12)}{d_\mu}-\frac{(n-1)(n-2)}{2}\frac{\chi^\alpha(12)}{d_\alpha}\right]\delta_{i_\alpha j_\alpha}, \tag{55}$$

where $i_\alpha, j_\alpha=1,\ldots,d_\alpha$.

We have one more summation rule, which plays a role of the standard orthogonality relation for irreps. Namely we have the following:

Proposition 17. *The PRIR* ϕ_R^μ *of* $S(n)$ *satisfies the following bilinear sum rule*

$$\forall\alpha,\beta,\gamma\in\mu \qquad \sum_{a=1}^{n}\sum_{k_\beta=1}^{d_\beta}(\phi_R^\mu)^{\alpha\beta}_{i_\alpha k_\beta}(an)(\phi_R^\mu)^{\beta\gamma}_{k_\beta j_\gamma}(an)=n\frac{d_\beta}{d_\mu}\delta^{\alpha\gamma}\delta_{i_\alpha j_\gamma}, \tag{56}$$

where α,β,γ *are irreps of* $S(n-1)$ *contained in the irrep* μ *of* $S(n)$.

Proof. The proof is based on the standard orthogonality relations for irreps, which in PRIR notation take the following form

$$\forall\alpha,\beta,\gamma\in\mu \qquad \sum_{\sigma\in S(n)}(\phi_R^\mu)^{\alpha\beta}_{i_\alpha k_\beta}(\sigma^{-1})(\phi_R^\mu)^{\beta\gamma}_{k_\beta j_\gamma}(\sigma)=\frac{n!}{d_\mu}\delta^{\alpha\gamma}\delta_{i_\alpha j_\gamma}, \tag{57}$$

for any irreps α,β,γ of the group $S(n-1)$ which are contained in the irrep μ of $S(n)$. On the other hand we may rewrite the *LHS* of the above equation as follows

$$LHS=\sum_{a=1}^{n}\sum_{\pi\in S(n-1)}\sum_{\xi,\theta\in\mu}\sum_{p_\xi,q_\theta}(\phi_R^\mu)^{\alpha\xi}_{i_\alpha p_\xi}(an)(\phi_R^\mu)^{\xi\beta}_{p_\xi k_\beta}(\pi^{-1})(\phi_R^\mu)^{\beta\theta}_{k_\beta q_\theta}(\pi)(\phi_R^\mu)^{\theta\gamma}_{q_\theta j_\gamma}(an). \tag{58}$$

Taking into account equation (43) we obtain

$$LHS=\sum_{a=1}^{n}\sum_{\pi\in S(n-1)}\sum_{p_\beta,q_\beta}(\phi_R^\mu)^{\alpha\beta}_{i_\alpha p_\beta}(an)\varphi^\beta{}_{p_\beta k_\beta}(\pi^{-1})\varphi^\beta_{k_\beta q_\beta}(\pi)(\phi_R^\mu)^{\beta\gamma}_{q_\beta j_\gamma}(an), \tag{59}$$

next applying the orthogonality relations for irreps φ^β of $S(n-1)$ we get

$$LHS=\frac{(n-1)!}{d_\beta}\sum_{a=1}^{n}\sum_{p_\beta}^{d_\beta}(\phi_R^\mu)^{\alpha\beta}_{i_\alpha p_\beta}(an)(\phi_R^\mu)^{\beta\gamma}_{p_\beta j_\gamma}(an). \tag{60}$$

Now comparing this with the *RHS* of the equation (57), we obtain the statement of the proposition. □

As a corollary from Propositions 14 and Proposition 15 we get

Corollary 18.

$$\forall\alpha\in\mu \quad \forall a=1,\ldots,n-1 \qquad \operatorname{Tr}\left[(\phi_R^\mu)^{\alpha\alpha}(an)\right]=\frac{n}{2}\frac{d_\alpha}{d_\beta}\chi^\mu(12)-\frac{n-2}{2}\chi^\alpha(12). \tag{61}$$

C Auxiliary facts concerning Young projectors

Let us define the following set of permutations

$$\Sigma_a = \{\sigma \in S(n-1) \,:\, \sigma(a) = n-1\}, \quad \text{then we have} \quad S(n-1) = \bigcup_{a=1}^{n-1} \Sigma_a. \tag{62}$$

Now we see that for every $\sigma \in \Sigma_a$ permutation $\sigma \circ (a, n-1)$ belongs to $S(n-2)$, since $(\sigma \circ (a, n-1))(n-1) = n-1$. Such property allows us to rewrite Young projectors P_μ, where $\mu \vdash n-1$ in a more convenient form, namely we have the following:

Fact 19. *Young projector P_μ, where $\mu \vdash n-1$ can be written as*

$$P_\mu = \frac{d_\mu}{(n-1)!} \sum_{a=1}^{n-1} \sum_{i,j=1}^{d_\mu} \varphi_{ij}^\mu(a, n-1) V(a, n-1) F_{ij}^\mu, \tag{63}$$

where

$$F_{ij}^\mu = \sum_{\pi \in S(n-2)} \varphi_{ji}^\mu \left(\pi^{-1}\right) V(\pi). \tag{64}$$

By $\varphi_{ij}^\mu(a, n-1), \varphi_{ji}^\mu\left(\pi^{-1}\right)$ we denote matrix elements of irreducible representations labelled by partition μ for the permutations $(a, n-1), \pi^{-1}$ respectively. Note that in the equation (64) we compute matrix elements of irreducible representations for partition $\mu \vdash n-1$, but over subgroup $S(n-2) \subset S(n-1)$.

Proof. Proof is based on straightforward calculations and observations summarized in the formula (62). We have the following chain of equalities:

$$\begin{aligned} P_\mu &= \frac{d_\mu}{(n-1)!} \sum_{\sigma \in S(n-1)} \chi^\mu\left(\sigma^{-1}\right) V(\sigma) = \frac{d_\mu}{(n-1)!} \sum_{a=1}^{n-1} \sum_{\pi \in S(n-2)} \chi^\mu\left((a, n-1) \circ \pi^{-1}\right) V\left((a, n-1) \circ \pi\right) \\ &= d_\mu \sum_{a=1}^{n-1} \sum_{i,j=1}^{d_\mu} \varphi_{ij}^\mu(a, n-1) V(a, n-1) \left(\frac{1}{(n-1)!} \sum_{\pi \in S(n-2)} \varphi_{ji}^\mu\left(\pi^{-1}\right) V(\pi) \right) \\ &= d_\mu \sum_{a=1}^{n-1} \sum_{i,j=1}^{d_\mu} \varphi_{ij}^\mu(a, n-1) V(a, n-1) F_{ij}^\mu. \end{aligned} \tag{65}$$

□

Every irreducible block labelled by $\mu \vdash n-1$ can be decomposed as a direct sum of smaller irreducible blocks labelled by partitions $\beta \vdash n-2$. Every such partition β is obtained by removing a single box from μ in the proper way. This togehter with the notion of PRIRs defined in Appendix B allows us to decompose every F_{ij}^μ from Fact 19 as

$$F_{ij}^\mu = \bigoplus_{\beta = \mu - \Box} \sum_{\pi \in S(n-2)} (\varphi_R^\mu)_{i_\beta j_\beta}^{\beta\beta} \left(\pi^{-1}\right) V(\pi). \tag{66}$$

Moreover, every operator F_{ij}^μ can be expressed in therms of the projectors E_{ij}^β as

$$F_{ij}^\mu = \bigoplus_{\beta = \mu - \Box} \frac{(n-2)!}{d_\beta} E_{i_\beta j_\beta}^\beta. \tag{67}$$

Fact 20. *Suppose that we are given an irreducible representation labelled by $\mu \vdash n-1$, then for every swap operator $V(k, n-1)$ between k^{th} and $(n-1)^{th}$ subsystem, and Young projector P_μ we have*

$$\sum_{k=1}^{n-1} V(k, n-1) P_\mu V(k, n-1) = (n-1) P_\mu. \tag{68}$$

Proof. We know that every Young projector associated with irreducible representation μ can be written as

$$P_\mu = \frac{d_\mu}{(n-1)!} \sum_{\sigma \in S(n-1)} \chi^\mu\left(\sigma^{-1}\right) V(\sigma), \tag{69}$$

where $\chi^\mu\left(\sigma^{-1}\right)$ is the character of irreducible representation μ calculated on the element $\sigma^{-1} \in S(n-1)$, and $V(\sigma)$ is the permutation operator acting on $\left(\mathbb{C}^d\right)^{\otimes(n-1)}$. Since operator P_μ belongs to the centre of the algebra $\mathbb{C}[S(n-1)]$ it commutes with all elements $V(\sigma) \in \mathbb{C}[S(n-1)]$, where $\sigma \in S(n-1)$ in particular with $V(k,n-1) \in \mathbb{C}[S(n-1)]$ for $k = 1, \dots, n-1$. This finishes the proof. □

Fact 21. *Let us denote by P_+ projector onto unnormalized maximally entangled state $|\psi^+\rangle = \sum_i |ii\rangle$ between $(n-1)^{th}$ and n^{th} subsystem, then:*

$$(\mathbf{1} \otimes P_+) V(k,n-1) (\mathbf{1} \otimes P_+) = \begin{cases} d(\mathbf{1} \otimes P_+) & \text{if} \quad k = n-1, \\ \mathbf{1} \otimes P_+ & \text{if} \quad k = 1, \dots, n-2. \end{cases} \tag{70}$$

In the above, by $V(k,n-1)$ we denote swap operator between k-th and $(n-1)$-th subsystem respectively, and by d dimension of the local Hilbert space.

Proof. For $k = n-1$ we have simply $(\mathbf{1} \otimes P_+)^2 = d(\mathbf{1} \otimes P_+)$, since P_+ is unnormalized. Now we have to prove the second case from the formula (70):

$$\begin{aligned} (\mathbf{1} \otimes P_+) V(k,n-1) (\mathbf{1} \otimes P_+) &= \left(\sum_{j_n, j_{n-1}=1}^{d} \mathbf{1}_1 \otimes \cdots \otimes \mathbf{1}_k \otimes \cdots \otimes |j_n\rangle\langle j_{n-1}| \otimes |j_n\rangle\langle j_{n-1}| \right) \\ &\times \left(\sum_{i_k, i_{n-1}=1}^{d} \mathbf{1}_1 \otimes \cdots \otimes \mathbf{1}_{k-1} \otimes |i_{n-1}\rangle\langle i_k| \otimes \cdots \otimes |i_k\rangle\langle i_{n-1}| \otimes \mathbf{1}_n \right) \\ &\times \left(\sum_{l_{n-1}, l_n=1}^{d} \mathbf{1}_1 \otimes \cdots \otimes \mathbf{1}_k \otimes \cdots \otimes |l_n\rangle\langle l_{n-1}| \otimes |l_n\rangle\langle l_{n-1}| \right) \end{aligned} \tag{71}$$

$$\begin{aligned} &= \sum_{\substack{j_n, j_{n-1}=1 \\ i_k, i_{n-1}=1 \\ l_{n-1}, l_n=1}}^{d} \mathbf{1}_1 \otimes \cdots \otimes \mathbf{1}_{k-1} \otimes |i_{n-1}\rangle\langle i_k| \otimes \cdots \otimes |j_n\rangle\langle j_{n-1}|i_k\rangle\langle i_{n-1}|l_n\rangle\langle l_{n-1}| \otimes |j_n\rangle\langle j_{n-1}|l_n\rangle\langle l_{n-1}| \\ &= \sum_{i_k, j_n, l_{n-1}=1}^{d} \mathbf{1}_1 \otimes \cdots \otimes \mathbf{1}_{k-1} \otimes |i_k\rangle\langle i_k| \otimes \cdots \otimes |j_n\rangle\langle l_{n-1}| \otimes |j_n\rangle\langle l_{n-1}| = \mathbf{1} \otimes P_+. \end{aligned} \tag{72}$$

□

Fact 22. *For an arbitrary element X of algebra $\mathscr{A}_n^{t_n}(d)$,* $\operatorname{Tr}_n X \in \mathbb{C}[S(n-1)]$.

Proof. From[18, 19] we know that algebra $\mathscr{A}_n^{t_n}(d)$ is spanned by the partially transposed permutation operators $V^{t_n}(\sigma)$, where $\sigma \in S(n)$. Let us take an arbitrary operator A defined on $n-1$ subsystems, then we can write

$$\operatorname{Tr}\left[V^{t_n}(\sigma) A \otimes \mathbf{1}_n\right] = \operatorname{Tr}\left[V(\sigma) A \otimes \mathbf{1}_n^{t_n}\right], \tag{73}$$

where $\mathbf{1}_n$ is the identity operator on last system, and t_n denotes standard transposition operation on last n-th system. We can now express the trace $\operatorname{Tr}_n[V^{t_n}(\sigma)] = \operatorname{Tr}[V(\sigma)]$, but $\operatorname{Tr}[V(\sigma)]$ for $\sigma \in S(n)$ belongs to $\mathbb{C}[S(n-1)]$, so we have $\operatorname{Tr}_n[V^{t_n}(\sigma)] \in \mathbb{C}[S(n-1)]$. □

D Proof of the auxiliary statements in Theorem 3

D.1 Proof of Eqn. (34)

Using the expression for P_μ from Fact 19 and applying Fact 21 we get:

$$\begin{aligned}
\zeta_{\mu,\mu'}(\alpha,\alpha') &= \sum_{a=1}^{n-1}\sum_{ij=1}^{d_\mu}\frac{d_\mu}{(n-1)!}\varphi_{ij}^{\mu}(a,n-1)\,\mathrm{Tr}\left[\left(P_\alpha F_{ij}^{\mu}\otimes\mathbf{1}\right)(\mathbf{1}\otimes P_+)V(a,n-1)(\mathbf{1}\otimes P_+)(P_{\alpha'}\otimes\mathbf{1})P_{\mu'}\right]\\
&= \sum_{a=1}^{n-1}\sum_{i,j=1}^{d_\mu}\frac{d_\mu d^{\delta_{a,n-1}}}{(n-1)!}\varphi_{ij}^{\mu}(a,n-1)\,\mathrm{Tr}\left[\left(P_\alpha F_{ij}^{\mu}P_{\alpha'}\otimes P_+\right)P_{\mu'}\right] \\
&= \sum_{a=1}^{n-1}\sum_{i,j=1}^{d_\mu}\frac{d_\mu d^{\delta_{a,n-1}}}{(n-1)!}\varphi_{ij}^{\mu}(a,n-1)\,\mathrm{Tr}\left[\left(P_\alpha F_{ij}^{\mu}P_{\alpha'}\otimes\mathbf{1}\right)P_{\mu'}\right].
\end{aligned} \tag{74}$$

In the last equality we have used the fact that $\mathrm{Tr}_n P^+ = \mathbf{1}$, where identity acts on $(n-1)$-th subsystem. Applying Fact 19 to operator $P_{\mu'}$ and calculating a partial trace over $(n-1)$-th subsystem we get:

$$\zeta_{\mu,\mu'}(\alpha,\alpha') = \sum_{a,b=1}^{n-1}\sum_{i,j=1}^{d_\mu}\frac{d_\mu d_{\mu'} d^{\delta_{a,n-1}} d^{\delta_{b,n-1}}}{[(n-1)!]^2}\varphi_{ij}^{\mu}(a,n-1)\varphi_{kl}^{\mu'}(b,n-1)\,\mathrm{Tr}\left[\left(P_\alpha F_{ij}^{\mu}\right)\left(P_{\alpha'}F_{kl}^{\mu'}\right)\right]. \tag{75}$$

D.2 Evaluating $g_{\mu,\mu'}(\alpha)$

$$g_{\mu,\mu'}(\alpha) = \sum_{a,b=1}^{n-1} d^{\delta_{a,n-1}} d^{\delta_{b,n-1}} \sum_{i_\alpha,j_\alpha=1}^{d_\alpha}(\phi_R^{\mu})_{i_\alpha j_\alpha}^{\alpha\alpha}(a,n-1)(\phi_R^{\mu'})_{j_\alpha i_\alpha}^{\alpha\alpha}(b,n-1), \tag{76}$$

with all irreps in the PRIR's form defined in Appendix B. Remarkably, this form allows us to directly evaluate these sums. First we partition the sums as follows:

$$\begin{aligned}
&\sum_{a,b=1}^{n-1} d^{\delta_{a,n-1}} d^{\delta_{b,n-1}} \sum_{i_\alpha,j_\alpha=1}^{d_\alpha}(\phi_R^{\mu})_{i_\alpha j_\alpha}^{\alpha\alpha}(an-1)(\phi_R^{\mu'})_{j_\alpha i_\alpha}^{\alpha\alpha}(bn-1)\\
&= \sum_{i_\alpha,j_\alpha=1}^{d_\alpha}\left\{\left[\sum_{a=1}^{n-1} d^{\delta_{a,n-1}}(\phi_R^{\mu})_{i_\alpha j_\alpha}^{\alpha\alpha}(a,n-1)\right]\left[\sum_{b=1}^{n-1} d^{\delta_{b,n-1}}(\phi_R^{\mu'})_{j_\alpha i_\alpha}^{\alpha\alpha}(b,n-1)\right]\right\}\\
&= \sum_{i_\alpha,j_\alpha=1}^{d_\alpha}\left\{\left[\sum_{a=1}^{n-2}(\phi_R^{\mu})_{i_\alpha j_\alpha}^{\alpha\alpha}(a,n-1)+d\delta_{i_\alpha j_\alpha}\right]\left[\sum_{b=1}^{n-2}(\phi_R^{\mu'})_{j_\alpha i_\alpha}^{\alpha\alpha}(b,n-1)+d\delta_{j_\alpha i_\alpha}\right]\right\}.
\end{aligned} \tag{77}$$

Now we use the second statement of Proposition 15 for $S(n-1)$, for the sums over a and b, which yields

$$\begin{aligned}
&\sum_{i_\alpha,j_\alpha=1}^{d_\alpha}\left\{\left[\left(\frac{(n-1)(n-2)}{2}\frac{\chi^{\mu}(12)}{d_\mu}-\frac{(n-2)(n-3)}{2}\frac{\chi^{\alpha}(12)}{d_\alpha}\right)\delta_{i_\alpha j_\alpha}+d\delta_{i_\alpha j_\alpha}\right]\times\right.\\
&\times\left.\left[\left(\frac{(n-1)(n-2)}{2}\frac{\chi^{\mu'}(12)}{d_{\mu'}}-\frac{(n-2)(n-3)}{2}\frac{\chi^{\alpha}(12)}{d_\alpha}\right)\delta_{j_\alpha i_\alpha}+d\delta_{j_\alpha i_\alpha}\right]\right\},
\end{aligned} \tag{78}$$

and after simple reordering we get

$$\begin{aligned}
&\left[\left(\frac{(n-1)(n-2)}{2}\frac{\chi^{\mu}(12)}{d_\mu}-\frac{(n-2)(n-3)}{2}\frac{\chi^{\alpha}(12)}{d_\alpha}\right)+d\right]\times\\
&\left[\left(\frac{(n-1)(n-2)}{2}\frac{\chi^{\mu'}(12)}{d_{\mu'}}-\frac{(n-2)(n-3)}{2}\frac{\chi^{\alpha}(12)}{d_\alpha}\right)+d\right]\sum_{i_\alpha,j_\alpha=1}^{d_\alpha}\delta_{i_\alpha j_\alpha}\delta_{j_\alpha i_\alpha}.
\end{aligned} \tag{79}$$

In equation (79) we recognize inside the square brackets the expression for eigenvalues $\gamma_\mu(\alpha)$ and $\gamma_{\mu'}(\alpha)$, this yields

$$\sum_{a,b=1}^{n-1} d^{\delta_{a,n-1}} d^{\delta_{b,n-1}} \sum_{i_\alpha, j_\alpha=1}^{d_\alpha} (\phi_R^\mu)^{\alpha\alpha}_{i_\alpha j_\alpha}(a,n-1)(\phi_R^{\mu'})^{\alpha\alpha}_{j_\alpha i_\alpha}(b,n-1) = \gamma_\mu(\alpha)\gamma_{\mu'}(\alpha) d_\alpha. \tag{80}$$

Substituting equation (80) into equation (36) we reduce expression for the fidelity F to:

$$F = \frac{1}{Nd^{N+2}} \sum_\alpha \frac{m_\alpha}{d_\alpha} \sum_{\substack{\mu\in\alpha \\ \mu'\in\alpha'}} d_\mu d_{\mu'} \sqrt{\gamma_\mu(\alpha)\gamma_{\mu'}(\alpha)}. \tag{81}$$

E Auxiliary facts concerning multiplicities of symmetric group

Let us consider the standard swap representation

$$V_n^d : S(n) \to \operatorname{Hom}\left[(\mathbb{C}^d)^{\otimes n}\right]. \tag{82}$$

It is well-known that it reduces to the form

$$V_n^d \cong \bigoplus_{\nu : h(\nu)\leq d} m_\nu \psi^\nu, \tag{83}$$

where ψ^ν are irreps of $S(n)$, m_ν their multiplicities, and by $h(\nu)$ we denote height of Young diagram ν. From theory of group characters we get

Proposition 23.

$$\sum_{\nu : h(\nu)\leq d} m_\nu^2 = \frac{1}{n!} \sum_{\sigma\in S(n)} d^{2l_n(\sigma)}, \tag{84}$$

where $l_n(\sigma)$ is the number of cycles in the permutation σ as a permutation of $S(n)$.

Now using the following

Lemma 24. *Let*

$$\sigma \in S(n) \wedge \sigma = (an)\rho : \rho \in S(n-1), \quad a = 1,\ldots,n, \tag{85}$$

then

$$l_n(\sigma) = \begin{cases} l_{n-1}(\rho) \ : \ a \neq n, \\ l_{n-1}(\rho)+1 \ : \ a = n \end{cases} \tag{86}$$

so for the non trivial cosets $S(n)/S(n-1)$ the number of cycles is the same.

We can now express the ratio of the multiplicities in closed form:

Proposition 25. *Let*

$$V_n^d \cong \bigoplus_{\nu : h(\nu)\leq d} m_\nu \psi^\nu, \qquad V_{n-1}^d \cong \bigoplus_{\alpha : h(\alpha)\leq d} m_\alpha \varphi^\alpha, \tag{87}$$

then

$$\frac{\sum_{\nu : h(\nu)\leq d} m_\nu^2}{\sum_{\alpha : h(\alpha)\leq d} m_\alpha^2} = \frac{d^2+n-1}{n}. \tag{88}$$

Lemma 26. *For any Young diagram $\mu : h(\mu) \leq d$ we have*

$$\frac{1}{m_\mu} \sum_{\alpha \in \mu} \gamma_\mu(\alpha) m_\alpha = n-1, \tag{89}$$

where $\alpha \in \mu$ denotes Young diagrams of $n-2$ which are obtained from Young diagrams of $n-1$ by removing one box in a proper way, and numbers $\gamma_\mu(\alpha)$ are given in Proposition 2.

Proof. Using the explicit form of the numbers $\gamma_\mu(\alpha)$ we have

$$\frac{1}{m_\mu} \sum_{\alpha \in \mu} \gamma_\mu(\alpha) m_\alpha = \frac{n-1}{m_\mu} \sum_{\alpha \in \mu} \frac{m_\mu d_\alpha}{m_\alpha d_\mu} m_\alpha = (n-1) \frac{1}{d_\mu} \sum_{\alpha \in \mu} d_\alpha. \tag{90}$$

The dimensions d_α corresponding to $\alpha \in \mu$ are precisely the dimensions of irreps of $S(n-2)$ appearing in the restriction of irreps of $S(n-1)$ to $S(n-2)$, so

$$\sum_{\alpha \in \mu} d_\alpha = d_\mu. \tag{91}$$

This finishes the proof. □

F Description of the operators E^α_{ij}

In this section we will briefly recall some properties of the algebra generated by a given complex finite dimensional representation of the finite group G. The content of this section can be found in standard textbooks on representation theory of finite groups and algebras, for example in[20].

Any complex finite-dimensional representation $D : G \rightarrow \mathrm{Hom}(V)$ of the finite group G, where V is a complex linear space, generates an algebra $A_V[G] \subset \mathrm{Hom}(V)$ which is isomorphic to the group algebra $\mathbb{C}[G]$ if the representation D is faithful:

$$A_V[G] = \mathrm{span}_{\mathbb{C}}\{D(g), \quad g \in G\}. \tag{92}$$

If the operators $D(g)$ are linearly independent, then they form a basis of the algebra $A_V[G]$ and $\dim A_V[G] = |G|$. It is also possible, using matrix irreducible representations, to construct a new basis which has remarkable properties, very useful in applications of representation theory. Below we describe this construction.

Notation 27. *Let G be a finite group of order $|G|$ which has r classes of conjugated elements. Then G has exactly r inequivalent, irreducible representations, in particular G has exactly r inequivalent, irreducible matrix representations. Let*

$$D^\alpha : G \rightarrow \mathrm{Hom}(V^\alpha), \qquad \alpha = 1, 2, \ldots, r, \qquad \dim V^\alpha = d_\alpha \tag{93}$$

be all inequivalent, irreducible representations of G and let us assume that these representations are all unitary (always possible) i.e.

$$D^\alpha(g) = (D^\alpha_{ij}(g)), \qquad \text{and} \qquad (D^\alpha_{ij}(g))^\dagger = (D^\alpha_{ij}(g))^{-1}, \tag{94}$$

where $i, j = 1, 2, \ldots, d_\alpha$.

The matrix elements $D^\alpha_{ij}(g)$ will play a crucial role in the following.

Definition 28. *Let $D : G \rightarrow \mathrm{Hom}(V)$ be an unitary representation of a finite group G such that the operators $D(g)$, $g \in G$ are linearly independent i.e. $\dim A_V[G] = |G|$ and let $D^\alpha : G \rightarrow \mathrm{Hom}(V^\alpha)$ be all inequivalent, irreducible representations of G described in Notation 27 above. Define*

$$E^\alpha_{ij} = \frac{d_\alpha}{|G|} \sum_{g \in G} D^\alpha_{ji}(g^{-1}) D(g), \tag{95}$$

where $\alpha = 1, 2, \ldots, r, \quad i, j = 1, 2, \ldots, d_\alpha, \quad E^\alpha_{ij} \in A_V[G] \subset \mathrm{Hom}(V)$.

The operators have the following properties:

Theorem 29. *There are exactly $|G|$ nonzero operators E^{α}_{ij} and*

$$D(g) = \sum_{ij\alpha} D^{\alpha}_{ij}(g) E^{\alpha}_{ij}. \tag{96}$$

2. *The operators E^{α}_{ij} are orthogonal with respect to the Hilbert-Schmidt scalar product in the space* $\mathrm{Hom}(V)$.

$$(E^{\alpha}_{ij}, E^{\beta}_{kl}) = \mathrm{Tr}\left[(E^{\alpha}_{ij})^{\dagger} E^{\beta}_{kl}\right] = m_{\alpha} \delta^{\alpha\beta} \delta_{ik} \delta_{jl}, \qquad m_{\alpha} \geq 1, \tag{97}$$

where m_{α} is equal to the multiplicity of the irreducible representation D^{α} in D and it does not depend on $i, j = 1, 2, \ldots, d_{\alpha}$.

3. *The operators E^{α}_{ij} satisfy the following composition rule*

$$E^{\alpha}_{ij} E^{\beta}_{kl} = \delta^{\alpha\beta} \delta_{jk} E^{\alpha}_{il}, \tag{98}$$

in particular E^{α}_{ii} are orthogonal projections.

Remark 30. *From item 2 of above theorem it follows that the expressions*

$$E^{\alpha}_{ij} = \frac{d_{\alpha}}{|G|} \sum_{g \in G} D^{\alpha}_{ji}(g^{-1}) D(g) \tag{99}$$

describe the transformation of orthogonalization of operators $D(g)$, $g \in G$ in the space $\mathrm{Hom}(V)$ *with the Hilbert-Schmidt scalar product.*

The operators E^{α}_{ii} are not only orthogonal projections onto their proper subspaces in V but they are also orthogonal with respect to the Hilbert-Schmidt scalar product in the space $\mathrm{Hom}(V)$. The basis $\{E^{\alpha}_{ij}\}$ plays essential role when $D : G \to \mathbb{C}[G]$ is the regular representation. In this case the properties of the basis $\{E^{\alpha}_{ij}\}$ expresses the well-known fact that the group algebra $\mathbb{C}[G]$ is a direct sum of simple matrix algebras generated by the irreducible representations of the group G. It is always possible to construct the operators E^{α}_{ij} even if the operators $D(g)$ are not linearly independent but in this case some of them will be zero.

G Proof of the SDP-related lemmas

G.1 Proof of Lemma 4:

Proof. The symmetries in our problem suggest that we may take $\Theta_{\overline{a}}$ as an element of the algebra $\mathbb{C}[S(n-2)]$. Thus, $\Theta_{\overline{a}} = \sum_{\alpha} x_{\alpha} P_{\alpha}$, where P_{α} are Young projectors and $x_{\alpha} \in \mathbb{R}_{+}$ which ensures that first constraint from (20) is automatically satisfied. Using this argumentation we can rewrite the second constraint from (20) restricted to an irrep labelled by $\alpha \vdash n-2$ as

$$\sum_{a=1}^{n-1} P^{+}_{a,n} \otimes \Theta_{\overline{a}}(\alpha) = x_{\alpha} \sum_{a=1}^{n-1} V(a,n-1) P^{+}_{n-1,n} \otimes P_{\alpha} V(a,n-1) = \frac{x_{\alpha}}{d} \eta(\alpha), \tag{100}$$

where $\eta(\alpha)$ are introduced in Theorem 1. Eigenvalues of the operator $\frac{1}{d}\eta(\alpha)$ are equal to $\frac{1}{d}\gamma_{\mu}(\alpha)$, where numbers $\gamma_{\mu}(\alpha)$ are eigenvalues of $\eta(\alpha)$ given in Theorem 2. To ensure that $\forall \alpha \ \frac{x_{\alpha}}{d}\eta(\alpha) \leq \mathbf{1}_{\alpha}$ we take

$$x_{\alpha} = \min_{\mu \in \alpha} \frac{1}{\frac{1}{d}\gamma_{\mu}(\alpha)} = d \min_{\mu \in \alpha} \frac{1}{\gamma_{\mu}(\alpha)}. \tag{101}$$

To obtain the minimum it suffices to insert $\gamma_{\mu^*}(\alpha)$, which is the maximal possible eigenvalue of the operator $\eta(\alpha)$ for some particular Young frame $\mu \vdash n-1$ obtained from $\alpha \vdash n-2$ by adding one box in the proper way. Inserting the optimal form of operators $\Theta_{\overline{a}}$ into equation (19), we get

$$p^{\star} = \frac{1}{d^{N+1}} \sum_{a=1}^{N} \mathrm{Tr}\left(\sum_{\alpha} x_{\alpha} P_{\alpha}\right) = \frac{N}{d^{N}} \sum_{\alpha} \frac{1}{\gamma_{\mu^*}(\alpha)} \mathrm{Tr}\, P_{\alpha} = \frac{N}{d^{N}} \sum_{\alpha} \frac{m_{\alpha} d_{\alpha}}{\gamma_{\mu^*}(\alpha)} = \frac{1}{d^{N}} \sum_{\alpha} m_{\alpha} d_{\alpha} \min_{\mu \in \alpha} \frac{1}{\gamma_{\mu}(\alpha)}. \tag{102}$$

□

G.2 Proof of Fact 5:

Proof. The operators $F_\mu(\alpha)$ are invariant under the action of $S(n-1)$ and thus under $S(n-2)$. The operator $V^{t_n}(n-1,n)$ is invariant under the action of $S(n-2)$. It follows that the composition $V^{t_n}(n-1,n)F_\mu(\alpha)$ is invariant under the action of $S(n-2)$. Moreover, using Fact 22 from Appendix C we have $\mathrm{Tr}_{n-1,n}\left[V^{t_n}(n-1,n)F_\mu(\alpha)\right] \in \mathbb{C}[S(n-2)]$, and since it is invariant under the action of $S(n-2)$, it must be of the form $\bigoplus_{\beta\vdash n-2} y(\beta)P_\beta$, where $y_\beta \in \mathbb{C}$. However, by Theorem 1 and Fact 13 from Appendix A we get

$$\begin{aligned} P_\beta V^{t_n}(n-1,n)F_\mu(\alpha) &= P_\beta V^{t_n}(n-1,n)M_\alpha P_\mu = P_\beta V^{t_n}(n-1,n)P_\alpha P_\mu \\ &= \delta_{\alpha\beta}P_\alpha V^{t_n}(n-1,n)F_\mu(\alpha). \end{aligned} \tag{103}$$

This implies that $\mathrm{Tr}_{n-1,n}\left[V^{t_n}(n-1,n)F_\mu(\alpha)\right] = y_\mu(\alpha)P_\alpha$, and thus

$$y_\mu(\alpha) = \frac{\mathrm{Tr}\left[V^{t_n}(n-1,n)F_\mu(\alpha)\right]}{d_\alpha m_\alpha} = \frac{\mathrm{Tr}\left[V^{t_n}(n-1,n)M_\alpha P_\mu\right]}{d_\alpha m_\alpha}. \tag{104}$$

To get the final result, we apply Fact 13 once more

$$y_\mu(\alpha) = \frac{\mathrm{Tr}\left[V^{t_n}(n-1,n)P_\alpha P_\mu\right]}{d_\alpha m_\alpha} = \frac{\mathrm{Tr}\left[P_\mu\left(P_\alpha\otimes\mathbf{1}\right)\right]}{d_\alpha m_\alpha} = \frac{m_\mu}{m_\alpha}. \tag{105}$$

□

G.3 Proof of Lemma 6:

Proof. Let us assume that the coefficients $x_{\mu^*}(\alpha)$ given in definition of the operator Ω in equation (23) are of the form $x_{\mu^*}(\alpha) = d\frac{m_\alpha}{m_{\mu^*}}$. One can easily see that $\Omega \geq 0$, and using Fact 5 we get

$$\begin{aligned} \mathrm{Tr}_{n-1,n}\left[P^+_{n-1,n}\Omega\right] &= \frac{1}{d}\mathrm{Tr}\left[V^{t_n}(n-1,n)\Omega\right] = \sum_\alpha \frac{m_\alpha}{m_{\mu^*}}\mathrm{Tr}\left[V^{t_n}(n-1,n)F_{\mu^*}(\alpha)\right] \\ &= \sum_\alpha P_\alpha = \mathbf{1}_{1,\dots,n-2}, \end{aligned} \tag{106}$$

where $\mathbf{1}_{1,\dots,n-2}$ denotes the identity operator defined on first $n-2$ subsystems. We thus see that the second constraint from expression (22) is also fulfilled. Finally we can calculate quantity $p_\star$ given in equation (21):

$$p_\star = \frac{1}{d^{N+1}}\mathrm{Tr}\,\Omega = \frac{1}{d^N}\sum_\alpha \frac{m_\alpha}{m_{\mu^*}}\mathrm{Tr}\,F_{\mu^*}(\alpha) = \frac{1}{d^N}\sum_\alpha m_\alpha^2\frac{d_{\mu^*}}{m_{\mu^*}} = \frac{1}{d^N}\sum_\alpha m_\alpha d_\alpha \min_{\mu\in\alpha}\frac{1}{\gamma_\mu(\alpha)}, \tag{107}$$

since $\mathrm{Tr}\,F_{\mu^*}(\alpha) = d_{\mu^*}m_\alpha$ by Theorem 1. □

G.4 Proof of Lemma 9

Proof. The symmetry of the problem suggests that optimal POVMs should be elements of the algebra $\mathbb{C}[S(n-2)]$. We represent them in the following form:

$$\forall\, a = 1,\dots,N \quad \Theta_{\overline{a}} = \sum_\alpha u(\alpha)P_{\alpha,\overline{a}}, \quad \text{with} \quad u(\alpha) = \frac{d^{N+1}g(N)m_\alpha}{Nd_\alpha}, \tag{108}$$

where $g(N) = 1/\sum_\nu m_\nu^2$ for all $\nu \vdash n-1$, and the above sum runs over all allowed irreps of $S(n-2)$. By $P_{\alpha,\overline{a}}$ we denote Young projectors onto irreps of $S(n-2)$, but defined on every subsystem except n-th and a-th. Since all coefficients $u(\alpha) \geq 0$, then the first constraint from (25) is satisfied. We choose the optimal state defined through X_A from (25) is of the form:

$$X_A = \sum_\mu c_\mu P_\mu, \quad \text{where} \quad c_\mu = \frac{d^N g(N)m_\mu}{d_\mu}, \tag{109}$$

where sum runs over all allowed irreps of $S(n-1)$. We see that

$$\operatorname{Tr} X_A = \sum_{\mu} c_\mu \operatorname{Tr} P_\mu = d^N \frac{\sum_\mu m_\mu^2}{\sum_\nu m_\nu^2} = d^N, \tag{110}$$

so the constraint on the trace of (25) is fulfilled. Moreover, we have that

$$\forall \mu \in \alpha \quad u(\alpha) = \frac{d}{\gamma_\mu(\alpha)} c_\mu, \tag{111}$$

where numbers $\gamma_\mu(\alpha)$ are eigenvalues given by Proposition 2. Thus, the second constraint from (25) is satisfied with the equality. For the above choices we compute the probability of success given in the statement of this Lemma plugging the choice of POVMs given in (108) into (24):

$$p^\star = \frac{1}{d^{N+1}} \sum_{a=1}^{N} \operatorname{Tr} \Theta_a = \frac{N}{d^{N+1}} \sum_\alpha u(\alpha) \operatorname{Tr} P_\alpha = \frac{\sum_\alpha m_\alpha^2}{\sum_\nu m_\nu^2} = \frac{N}{N+d^2-1} = 1 - \frac{d^2-1}{N+d^2-1}, \tag{112}$$

where we used the fact that $\operatorname{Tr} P_\alpha = m_\alpha d_\alpha$, and plugged the ratio from Proposition 25 in Appendix E. □

G.5 Proof of Lemma 10

Proof. Since the operators $F_\mu(\alpha)$ are invariant under the action of $S(n-1)$ and $\operatorname{Tr}_n F_\mu(\alpha) \in \mathbb{C}[S(n-1)]$, the partial trace decomposes as $\operatorname{Tr}_n F_\mu(\alpha) = \bigoplus_{\mu \vdash n-1} a_\nu P_\nu$, where $a_\nu \in \mathbb{C}$. On the other hand, by Theorem 1 we have

$$P_\nu F_\mu(\alpha) = P_\nu M_\alpha P_\mu = \delta_{\mu\nu} F_\mu(\alpha), \tag{113}$$

hence

$$\operatorname{Tr}_n F_\mu(\alpha) = a_\mu P_\mu, \tag{114}$$

and

$$a_\mu = \frac{\operatorname{Tr} F_\mu(\alpha)}{\operatorname{Tr} P_\mu} = \frac{m_\alpha d_\mu}{m_\mu d_\mu} = \frac{m_\alpha}{m_\mu}, \tag{115}$$

where we use that $\operatorname{Tr} F_\mu(\alpha) = m_\alpha d_\mu$ from Theorem 1. □

G.6 Proof of Lemma 11

Proof. We represent the coefficients $x_\mu(\alpha)$ of Ω from (29) as follows:

$$x_\mu(\alpha) = \frac{d}{d^2+N-1} \gamma_\mu(\alpha), \tag{116}$$

where numbers $\gamma_\mu(\alpha)$ are eigenvalues of the PBT operator η given in Proposition 2. Making use of Theorem 1 we get

$$\Omega = \sum_\alpha \sum_{\mu \in \alpha} x_\mu(\alpha) F_\mu(\alpha) = \frac{d}{N+d^2-1} \eta \geq 0 \tag{117}$$

for any value d and N, so the first constraint from (28) is fulfilled, since $\eta \geq 0$. For the second constraint, making use of the symmetry of the problem it suffices to estimate it for $a = n-1$:

$$\operatorname{Tr}_{n-1,n}\left[P^+_{n-1,n}\Omega\right] = \frac{1}{N+d^2-1} \operatorname{Tr}_{n-1,n}\left[V^{t_n}(n-1,n)\eta\right] = \mathbf{1}_{1\ldots n-2}, \tag{118}$$

where $\mathbf{1}_{1\ldots n-2}$ denotes the identity operator acting on $n-2$ subsystems. Finally we need $\operatorname{Tr}_n \Omega$:

$$\operatorname{Tr}_n \Omega = \frac{d}{N+d^2-1} \sum_\alpha \sum_{\mu \in \alpha} \gamma_\mu(\alpha) \operatorname{Tr}_n F_\mu(\alpha). \tag{119}$$

Using Lemma 10 we reduce equation (119) to

$$\operatorname{Tr}_n \Omega = \frac{d}{N+d^2-1}\sum_{\alpha}\sum_{\mu\in\alpha}\gamma_\mu(\alpha)\frac{m_\alpha}{m_\mu}P_\mu = \frac{d}{N+d^2-1}\sum_{\mu}\frac{1}{m_\mu}\sum_{\alpha\in\mu}\gamma_\mu(\alpha)m_\alpha P_\mu, \tag{120}$$

where by $\alpha \in \mu$ we denote Young diagrams α of $n-2$ which can be obtained from Young diagrams μ of $n-1$ by removing one box in a proper way. Now using the explicit form of $\gamma_\mu(\alpha)$ given in Proposition 2 and Lemma 26 from Appendix E we can simplify Eqn. (120)

$$\operatorname{Tr}_n \Omega = \frac{dN}{N+d^2-1}\sum_{\mu}\frac{1}{d_\mu}\sum_{\alpha\in\mu}d_\alpha P_\mu = \frac{dN}{N+d^2-1}\sum_{\mu}P_\mu = \frac{dN}{N+d^2-1}\mathbf{1}_{1\ldots n-1}, \tag{121}$$

where $\mathbf{1}_{1\ldots n-1}$ is identity operator defined on $n-1$ subsystems. Now taking $b = \frac{1}{d^N}\frac{N}{N+d^2-1}$, we satisfy the third constraint from (28)

$$p_\star = d^N b = 1 - \frac{d^2-1}{N+d^2-1} = p^\star. \tag{122}$$

□
